# Exploring Health Educational Interventions for Children With Congenital Heart Disease: Scoping Review

**DOI:** 10.2196/64814

**Published:** 2025-01-24

**Authors:** Neda Barbazi, Ji Youn Shin, Gurumurthy Hiremath, Carlye Anne Lauff

**Affiliations:** 1 Department of Design Innovation, College of Design University of Minnesota, Twin Cities Minneapolis, MN United States; 2 Division of Pediatric Cardiology, Department of Pediatrics Medical School University of Minnesota, Twin Cities Minneapolis, MN United States

**Keywords:** congenital heart disease, children health literacy, health education, health education interventions, patient-centered care, design, pediatric, PRISMA

## Abstract

**Background:**

Congenital heart disease (CHD) is the most common birth defect, affecting 40,000 births annually in the United States. Despite advances in medical care, CHD is often a chronic condition requiring continuous management and education. Effective care management depends on children’s understanding of their condition. This highlights the need for targeted health educational interventions to enhance health literacy among children with CHD.

**Objective:**

This scoping review aims to map and explore existing health educational interventions for children with CHD. The review identifies the types of interventions, target populations, delivery methods, and assessed outcomes. The goal is to consolidate fragmented research, identify gaps, and establish future research agendas.

**Methods:**

Comprehensive searches were conducted in February 2024 using the PRISMA-ScR (Preferred Reporting Items for Systematic Reviews and Meta-Analyses Extension for Scoping Reviews) framework across multiple databases: APA PsycINFO, MedlinePlus via Ovid, Web of Science, ACM Digital Library, Scopus, and EBSCOhost (CINAHL Complete, CINAHL Ultimate, Health Source: Nursing/Academic Edition, and ERIC). The search covered health care, design, and human-computer interaction disciplines to capture the interdisciplinary nature of CHD health educational interventions. There was no predefined time limit due to the limited number of relevant studies. Eligible studies were in English, published in peer-reviewed journals, and focused on primary data about educational health interventions for children with CHD. We extracted and synthesized data using thematic analysis.

**Results:**

The review identified 11 studies: 9 randomized controlled trials and 2 observational studies. These used 6 educational strategies: 3D patient-specific models (n=3), habit formation interventions (n=2), empowerment-based health education programs (n=2), rehabilitation interventions (n=2), web-based portals (n=1), and videotape presentations (n=1). Interventions ranged from brief outpatient sessions to 1.5-year programs, with follow-up from none to 24 months. Studies aimed to improve coping, self-management, and knowledge for children with CHD and their families. The most frequently used assessment method was the independent samples *t* test (n=4) for pre- and postassessments, and all 11 studies used questionnaires, 8 of which incorporated qualitative feedback. The target participants for these interventions were children aged 13 years and older (n=3), parents (n=2), and children of various ages and their parents (n=6). Outcomes included improved children’s health literacy, reduced parental burden, and increased health care provider efficiency.

**Conclusions:**

This review underscores the critical need for tailored educational interventions for children with CHD. Current research mainly focuses on adolescents and relies heavily on parental involvement, possibly overlooking the specific needs of younger children younger than 13 years of age. It is essential to develop engaging, age-appropriate interventions that actively involve children with CHD in their health care journey. Effective health educational interventions are crucial in empowering these young patients and improving their long-term health outcomes.

## Introduction

### Background

**Figure 1:**
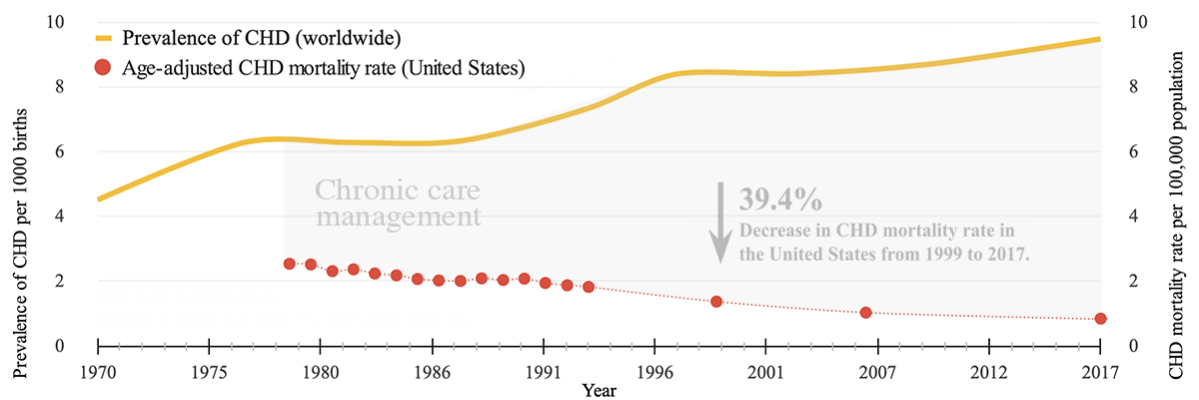
Worldwide CHD prevalence and the effect of US survival rates on care management. Data were sourced from Boneva et al [8], Liu et al [7], Lopez et al [6]. CHD: congenital heart disease.

Congenital heart disease (CHD) is a structural abnormality in the heart present at birth. It is the most common birth defect, affecting 1.35 million newborns worldwide and around 40,000 births annually in the United States. CHD often leads to other health complications and poses lifelong challenges to affected children, families, and health care systems [1-3]. Improved medical and surgical care have substantially increased survival rates, with up to 90% of children with CHD now surviving through to adulthood [4,5]. Despite improved medical care, CHD is more like a chronic condition that requires early diagnosis, timely treatment, and ongoing management. Figure 1 shows the worldwide prevalence of CHD from 1970 to 2017 and how survival rates in the United States since 1999 impact the need for continuing care [2,5-8]. Effective long-term care requires a comprehensive understanding of the condition by both pediatric patients and caregivers. Traditionally, health care providers have relied on parents to educate their children about their condition, assuming effective transmission of information. This approach often falls short, with parents struggling to comprehend and recall the information provided. Insufficient knowledge leads to extensive education during appointments, causing confusion and anxiety for children with CHD and their families [9-12]. Without proper education, the ability to proactively manage the condition diminishes, potentially leading to worse health outcomes, greater difficulty transitioning to adult care, and increased hospitalizations [4,13]. Accessible health information and organized educational support systems are crucial for improving health education, self-management skills, and health literacy (HL) among pediatric patients and caregivers [2,14].

### Prior Work

HL is defined as “the degree to which individuals have the capacity to obtain, process, and understand basic health information and services needed to make appropriate health decisions” [9]. However, there are uncertainties about the best educational and support approaches for improving HL in pediatric patients with CHD. Learning about this complex subject at a young age presents significant challenges. Limited learning opportunities hinder adequate information delivery and understanding [10]. Moreover, current educational materials (eg, pamphlets and postvisit summaries) are often inappropriate for young children and are primarily intended for their caregivers or parents [10,12]. To tackle these challenges, health educational interventions are designed to enhance individuals’ knowledge, attitudes, skills, and behaviors to manage their condition [15]. These interventions are crucial for empowering children with CHD to understand their condition, adhere to treatment plans, and navigate the health care system effectively.

Recent focus has highlighted the importance of involving children with CHD and their caregivers in developing and executing their care plans through health educational interventions [10,12]. Despite this, the literature on educational interventions for children with CHD remains limited. To our knowledge, no systematic or scoping reviews have been conducted on this topic. Only a few scoping and systematic studies have attempted to understand the experiences of children with CHD, their families, and health care providers in managing CHD [2,16-18]. Furthermore, some studies focus on the coping mechanisms of parents and families of children with CHD rather than on the children themselves [19-21]. Therefore, a scoping review is necessary to consolidate fragmented research, identify existing gaps, and establish a future research agenda [22].

### Purpose and Objectives

This scoping review systematically maps and explores existing educational interventions for children with CHD to identify intervention types, target populations, delivery methods, and assessed outcomes. Textbox 1 presents the PICOTS (population, intervention, comparator, outcome, timing, and setting) framework for educational interventions for children with CHD. PICOTS helps clarify and organize research questions. The key questions (KQs) addressed are:

KQ1: What types of educational interventions are available for children with CHD?

KQ2: What are the study designs, target populations, and the roles of various stakeholders in these educational interventions?

KQ3: What outcomes are assessed, and what approaches are used for assessment?

KQ4: What gaps exist in the current literature regarding educational interventions for children with CHD, and what areas require further exploration?

**Figure 2:**
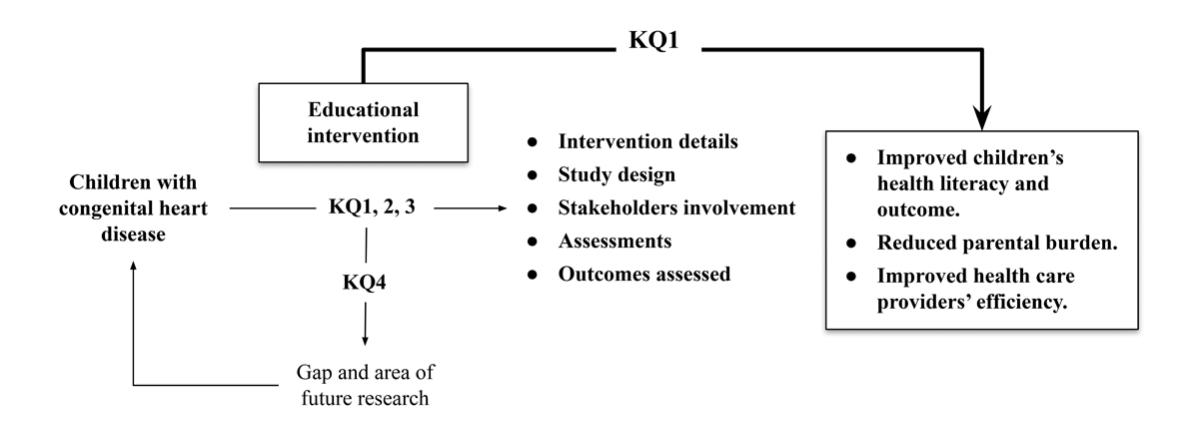
Logic model for the PICOTS (population, intervention, comparator, outcome, timing, and setting) framework and 4 key questions (KQs).

Figure 2 presents the analytical framework for the scoping review. It outlines a structured approach to address KQs concerning CHD and educational interventions for pediatric patients. The review covers intermediate outcomes, including intervention details, study design, stakeholder involvement, and assessed outcomes. The primary outcomes are improved children’s HL, reduced parental burden, and enhanced efficiency of health care providers. Suboutcomes related to children’s HL enhancement include understanding CHD, self-management or habits, coping or quality of life, empowerment, health care use, and health outcomes. The review also focuses on providing educational, emotional, caregiving, and financial support to reduce parental burden. Additionally, it considers suboutcomes related to saving time or effort, treatment adherence, care coordination, shared decision-making, and patient or family satisfaction to enhance the efficiency of health care providers.

**PICOTS (population, intervention, comparator, outcome, timing, and setting) framework for key questions on health educational interventions for children with congenital heart disease (CHD).**

**Population**

- Children diagnosed with CHD <18 years.

**Intervention**

- Educational interventions targeting health literacy improvement among children with CHD.

**Comparators**

- Any comparator (as this is a scoping review).

**Outcomes**

- Engagement (use and satisfaction).
- Improved health literacy in children with CHD (understanding of CHD, self-management or habits, coping or quality of life, empowerment, health care use, and health outcome).
- Reduced parental burden through effective educational support (educational support, emotional support, and financial support).
- Enhanced health care efficiency by minimizing education needs during medical appointments (saving time or effort, treatment adherence, care coordination, shared decision-making, and patient or family satisfaction).

**Timing**

- No restrictions.

**Setting**

- All types of studies (as this is a scoping review), including various health care (eg, hospitals and clinics), nonhealth care (eg, school and support community), and home settings.

## Methods

### Literature Search Strategy

This study used a scoping approach guided by the PRISMA-ScR (Preferred Reporting Items for Systematic Reviews and Meta-Analyses Extension for Scoping Reviews) framework [23]. Scoping reviews provide an overview of emerging evidence without aiming to appraise and synthesize results for a specific question. They are useful when it is still unclear what specific research questions a systematic review can address. Additionally, scoping reviews help inform and identify current research practices and methodologies in emerging research fields [22-25]. This research process was structured using the PRISMA (Preferred Reporting Items for Systematic Reviews and Meta-Analyses) framework, including four stages: (1) identification of relevant literature searches on various databases using specific keywords, (2) screening the title and abstract of the identified studies, (3) eligibility—full-text review of screened results to eliminate studies outside our intended scope, and (4) inclusion, helped extract relevant data that met the defined criteria [26]. The PRISMA-ScR checklist is available in Multimedia Appendix 1.

### Eligibility Criteria

To be included in the review, studies must be written in English and published in a peer-reviewed journal to ensure accessibility and credibility. We only considered studies with a methods section, focusing on primary data and analyses rather than studies like systematic reviews. Our focus was on CHD in pediatric patients younger than 18 years of age, and the studies addressed educational interventions for this demographic, not just HL. We excluded studies that solely targeted parents or caregivers, focused only on health care providers, or related to transition care from pediatric to adult health care, as these areas fall outside our specific review scope. However, we included studies involving parents only when their participation aimed to improve the child’s health outcomes, focusing on enhancing the children’s educational experience rather than the parents. This selection process ensured that the included studies were directly relevant to pediatric CHD and educational health interventions.

### Information Sources and Search

We conducted comprehensive searches across the following bibliographic databases during February 2024 to identify potentially relevant studies without a predefined time limit. Given the initial research indicating a scarcity of relevant studies, we maintained an open search time frame. We gathered relevant studies from APA PsycINFO and MedlinePlus via Ovid, Web of Science, ACM Digital Library, Scopus, and EBSCOhost, including CINAHL Complete, CINAHL Ultimate, Health Source: Nursing/Academic Edition, and ERIC. These databases cover health care, psychology, education, design, and human-computer interaction disciplines. They were selected to reflect the interdisciplinary nature of CHD health educational interventions.

Three main concepts were identified based on the research questions: HL, pediatric, and CHD. Synonyms and related concepts were incorporated to ensure a comprehensive search for related terminologies used in the literature. For example, synonyms of “pediatric” included “child,” “children,” “toddler,” and “preschool,” while related concepts like “boy” and “girl” were also included. Given the cross-disciplinary database search, listing each concept’s relevant ideas and terms was important. MeSH terms and keywords were tailored to each database’s specifications, as detailed in Multimedia Appendix 2. Boolean search strings were formulated using the OR operator for synonyms of the main concepts (ie, “children” OR “child” OR “pediatric”) and the AND operator to combine the 3 main concepts (ie, “health literacy” AND “congenital heart disease” AND “pediatric”). The terms were refined through multiple iterations by adding new terms and synonyms or adjusting specificity to enhance the quality of results. For instance, recognizing that the term “health literacy” may be omitted in studies involving health education interventions, alternative terms such as “healthcare knowledge,” “health awareness,” and “health education” were included. Additionally, we intentionally avoided using “intervention” as a synonym for “health literacy” to ensure that studies focusing on clinical and surgical interventions in CHD were not included (Table 1).

**Table 1:** Boolean search strings in categories.

Categories	AND or NOT	Boolean search string
Health literacy	AND	(“Health Literacy” OR “Healthcare Literacy” OR “Medical Literacy” OR “Health Understanding” OR “Health Education” OR “Healthcare Education” OR “Health Information Literacy” OR “Medical Comprehension” OR “Healthcare Knowledge” OR “Health Knowledge” OR “Health Proficiency” OR “Health Awareness” OR “Medical Awareness” OR “Health Competency” OR “Health Communication” OR “Information Literacy” OR “Patient Education” OR “Health Promotion” OR “Health Teaching”)
Pediatric	AND	(Children OR Child* OR Kid OR Kids OR Girl* OR Boy OR Boys* OR Toddler* OR Childhood OR Preschool* OR Pre-school* OR Kindergarten* OR School OR Minors OR Pediatric* OR Paediatric*)
Congenital heart disease	AND	(“Congenital Heart Defects” OR “Child Heart Disease” OR “Heart Defects” OR “Congenital Heart Disease” OR “Pediatric Cardiology” OR “Paediatric Cardiology” OR “Cardiac Defect*”)

The first author (NB) and a collaborator independently screened the titles and abstracts using the PICO Portal, reaching a consensus on selections for full-text screening and consulting the health librarian for final decisions in disagreements. Before and during the screening process, the first (NB), second (JYS), and last authors (CAL) held weekly meetings to clarify the selection of databases, concepts, and criteria as well as to draft an internal guideline. For example, following our discussions, we added the ACM Digital Library database to broaden our search to include technology-mediated solutions. These meetings continued during data charting and thematic analysis. We also sought guidance from a medical expert, a public health expert, and 2 librarians throughout the review. We used Zotero (Corporation for Digital Scholarship) for reference management and Google Sheets and Microsoft Excel for data abstraction. We established decision rules to guide the coding process and ensure consistency in cases requiring subjective interpretation.

The research team abstracted data on study characteristics: (1) study identification (ie, ID, author or year, country, title, study design, date of study, setting, and objective or purpose), (2) participant details (ie, target population, intervention tested on, sample size, demographic, inclusion or exclusion criteria, and CHD severity), (3) intervention specification (ie, intervention, format, description, comparison, stakeholders’ roles, duration, and follow-up), and (4) outcome specification (ie, outcome measures, results, and statistical analysis). Abstraction tables are shown in Tables S1-S5 in Multimedia Appendix 3 [27-37].

### Data Synthesis

Due to the heterogeneous nature of the data, the research team used a qualitative approach using affinity diagramming and thematic analysis [38,39]. This approach enabled us to explore the data without predetermined frames and uncover emerging themes to answer our scoping review questions and objectives. We began by categorizing the studies based on the types of interventions they covered. We then performed open coding on all 11 papers to identify the specifications of educational interventions (ie, objectives, strategies, stakeholder involvement, and outcomes) for children with CHD. Through constant comparison and iterative coding, thematic categories emerged. Initially, open coding of studies yielded 43 discrete codes. Subsequently, these codes were iteratively aggregated based on commonalities, resulting in 15 representative codes. Next, affinity diagramming was used to cluster these 15 codes according to similarity, difference, and hierarchy relationships.

This process allowed us to establish high-level themes, refined through constant comparison and iterative coding. The key themes that emerged from the data include (1) types of educational interventions, (2) study design and stakeholder involvement, and (3) evaluation methods and outcome objectives. Each main theme was further divided into 2 subthemes (Textbox 2). This thematic synthesis provided a clear and structured understanding of the educational interventions for children with CHD, covering their implementation and evaluation.

**Identified themes and subthemes.**

**Types of educational interventions**

- Educational strategies and objectives (eg, engaging sessions and disease education)
- Intervention duration (eg, duration and frequency of educational interventions)

**Study design and stakeholder involvement**

- Study design (eg, observational and randomized controlled trial)
- Target age groups and stakeholder involvement (eg, children and parents)

**Evaluation methods and outcome objectives**

- Assessed outcomes (eg, health literacy and health outcome)
- Data collection and analysis techniques (eg, questionnaires and interviews)

## Results

### Selection of Sources of Evidence

**Figure 3:**
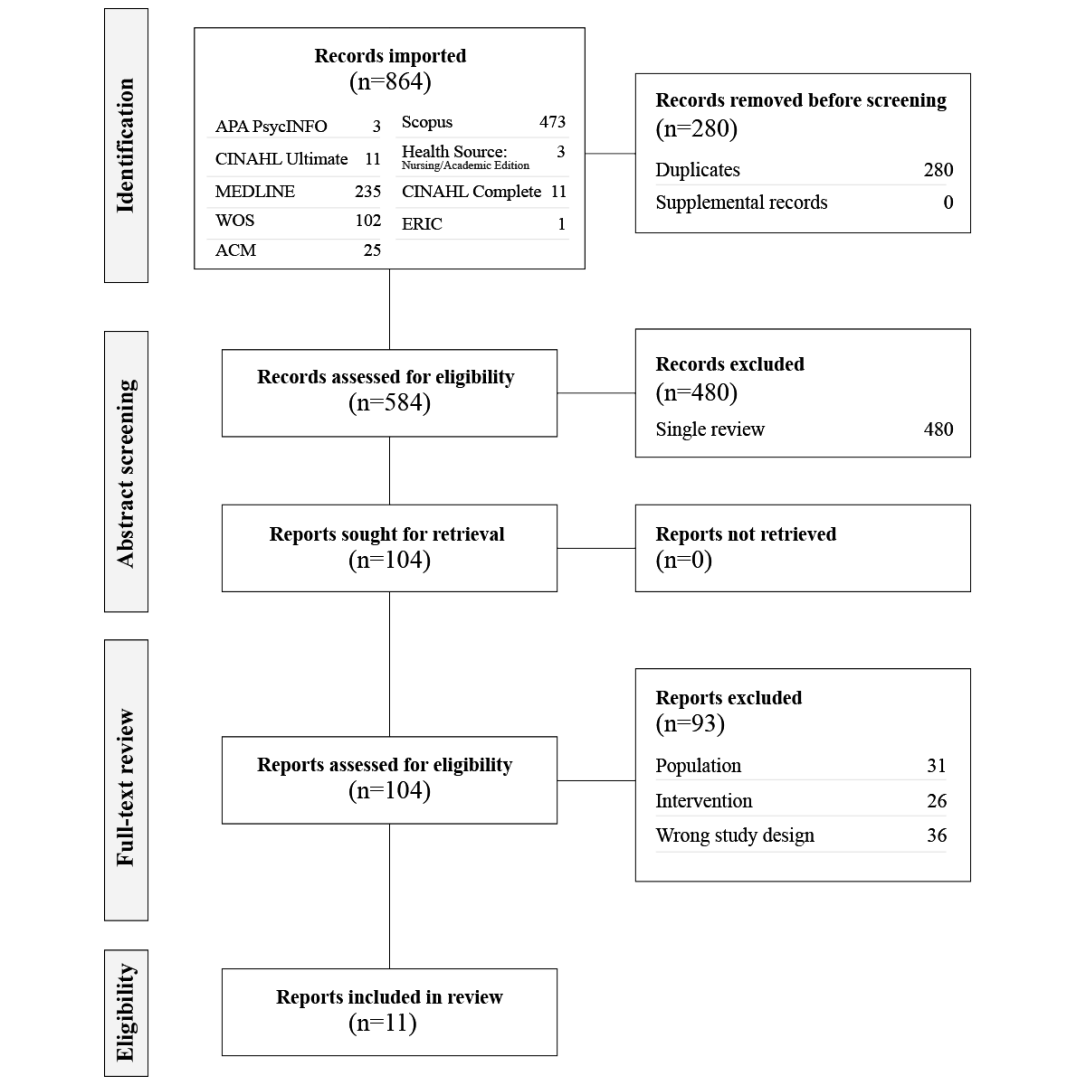
PRISMA (Preferred Reporting Items for Systematic Reviews and Meta-Analyses) diagram of the literature review process.

The literature search identified 864 records across 5 databases: APA PsycINFO (n=3), MEDLINE (n=235), Web of Science (n=102), ACM Digital Library (n=25), Scopus (n=473), CINAHL Complete (n=11), CINAHL Ultimate (n=11), Health Source: Nursing/Academic Edition (n=3), and ERIC (n=1). After removing 280 duplicates and supplemental materials, 584 records remained for abstract screening. Following abstract and title screening by the research team, 480 records were excluded for not meeting the review criteria. The full texts of the remaining 104 reports were assessed for eligibility. Of these, 93 reports were excluded for various reasons: 31 due to the population mismatch (ie, focusing on parents or caregivers or health care providers, participants aged >18 years, or studying general heart disease), 26 because the intervention was not relevant (ie, no interventions implemented, assessments used as interventions, or noneducational interventions), and 36 were excluded based on the wrong study design (ie, focusing on transitions to adulthood or objectives aimed at parents or health care providers). Finally, 11 reports met all the inclusion criteria and were selected for inclusion in the scoping review. Figure 3 illustrates the results of the literature search and screening. We used the PICO Portal review software to support the screening process. The reasons for exclusion at each stage were clearly documented, ensuring transparency and adherence to the PRISMA guidelines. References for papers excluded in the full-text review can be found in Multimedia Appendix 4.

### Characteristics of Sources Evidence

**Figure 4:**
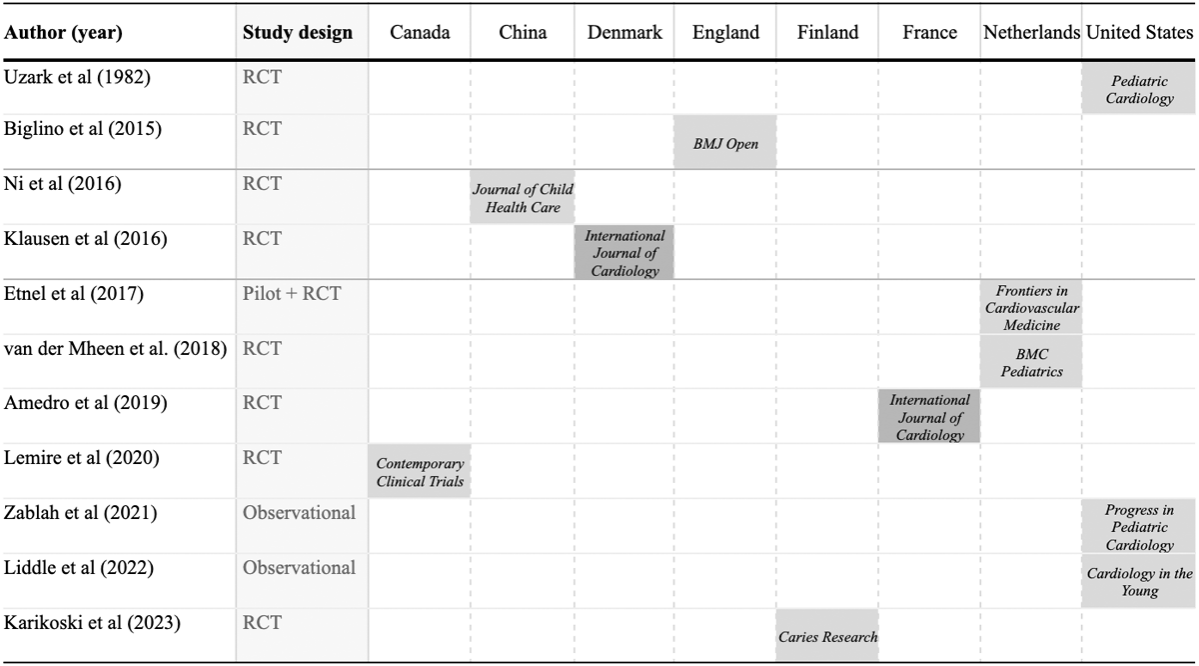
Characteristics of included studies [27-37]. RCT: randomized controlled trial.

From 1982 to 2023, 11 studies were reviewed, focusing on educational interventions for pediatric patients with CHD [27-37] (Figure 4). There was a gap in the selected studies from 1982 to 2015. After that, 1 study was published every year until 2023, except for 2016, which had 2 studies [31,34]. Two studies were from the Netherlands in 2017 and 2018 [29,36], while 3 were from the United States in 1982, 2021, and 2022 [33,35,37]. Among these studies were 9 randomized controlled trials (RCTs) [27-32,34-36], with 1 covering both a pilot and RCT [29]. Additionally, 2 were observational studies [33,37]. Even though we searched design, human-computer interaction, and health databases, the studies were published in 9 medical and health-related journals. Two of the reviewed studies were published in the *International Journal of Cardiology* [27,31].

### Results of Individual Sources of Evidence

#### Overview

In exploring educational interventions for children with CHD, the first emergent theme is “types of educational interventions,” with subthemes of “educational strategies and objectives” and “intervention duration.” Educational strategies and objectives refer to the strategies used to support children with CHD and how these interventions address specific objectives, like disease education. Duration presents the timing and duration of these interventions, whether they are 1-time sessions, have follow-ups, or are repeated over time. This theme and its subthemes address KQ1: What types of educational interventions are available for children with CHD?

#### Educational Strategies and Objectives

**Figure 5:**
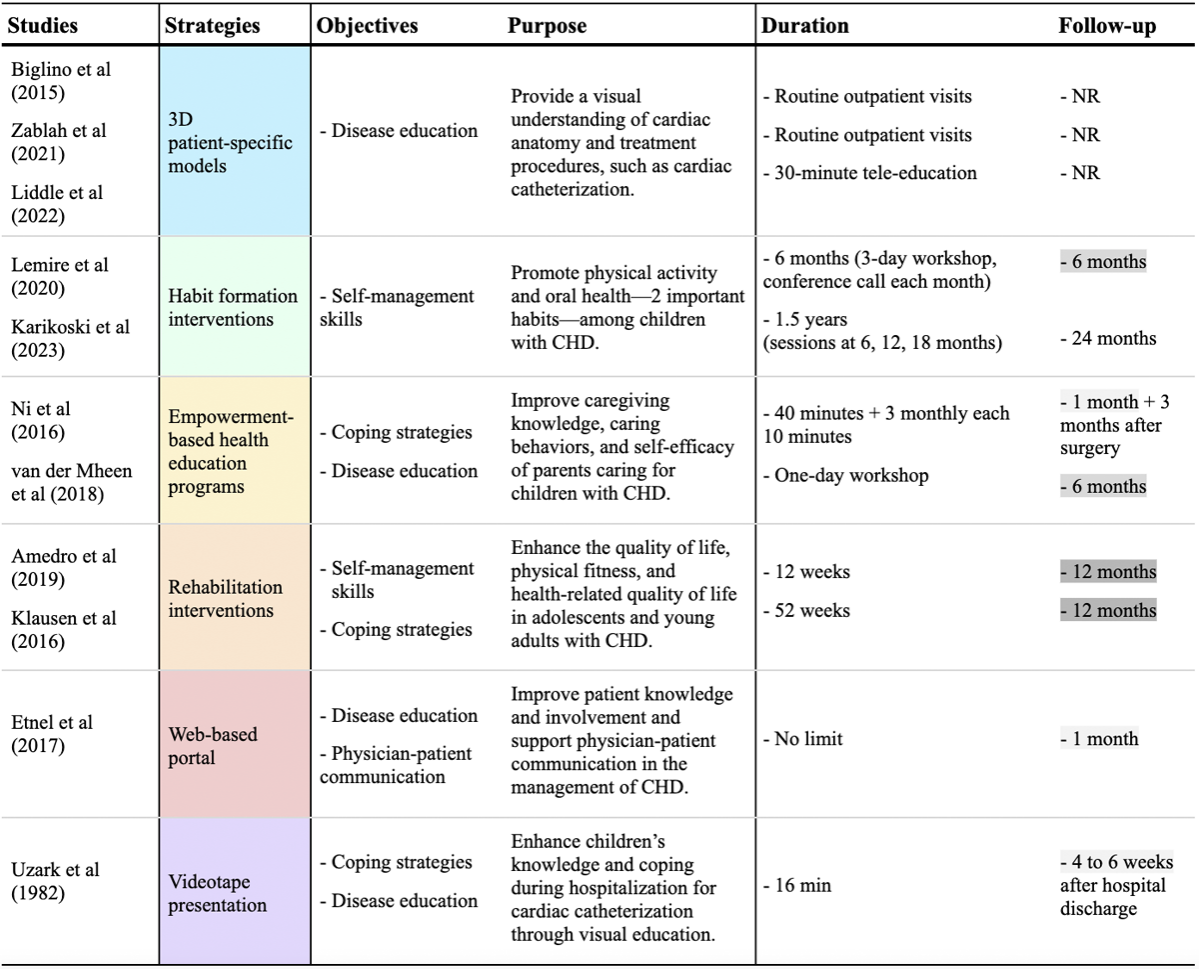
Educational strategies, objectives, and intervention durations [27-37]. CHD: congenital heart disease; NR: not reported.

Across the reviewed studies, 6 educational strategies were used to support children with CHD and their families (Figure 5). These strategies include the use of 3D patient-specific models, habit formation interventions, empowerment-based health education programs, rehabilitation interventions, a web-based portal, and videotape presentations.

3D patient-specific models are anatomical models created from medical imaging data like computed tomography or magnetic resonance imaging scans, providing a detailed representation of a child’s heart. They help visualize and understand complex cardiac structures and treatment plans. Three studies used these models to educate about cardiac anatomy and treatment plans. These models were accompanied by engaging discussions and supplemented with diagrams or images, clarifying complex anatomical structures and treatment options. One of these studies specifically targeted adolescent patients, combining 3D models with tele-education. These interventions offered a visual understanding of the heart’s structure and treatment procedures, including cardiac catheterization [28,33,37]. Habit formation interventions aim to instill healthy habits in children with CHD through structured programs. These interventions often involve multiple components, such as printed materials, toolkits, websites, and counseling sessions, to encourage and support the development of these beneficial habits. Two studies focused on these interventions. One aimed at promoting physical activity through a multifaceted intervention, including printed materials, a physical activity toolkit, and a website. The other focused on oral health promotion through counseling, distributing oral hygiene products, and providing written information [30,32].

Empowerment-based health education programs empower parents and family members by enhancing their knowledge, caregiving behaviors, and self-efficacy in managing a child’s CHD. They typically include face-to-face education sessions, workshops, and follow-up support such as telephone calls, helping caregivers feel more confident and competent in their roles. Two studies implemented these programs to improve the caregiving knowledge, caring behaviors, and self-efficacy of parents or family members caring for children with CHD. One offered face-to-face education sessions and follow-up telephone calls, while the other conducted workshops [34,36]. Rehabilitation interventions aim to enhance the physical and psychological well-being of children and adolescents with CHD. These include structured cardiac rehabilitation programs (center or home-based) and eHealth interventions that use technology to improve physical fitness, activity levels, and overall health-related quality of life over an extended period. Two studies explored these interventions. One evaluated the impact of a combined center- and home-based cardiac rehabilitation program on the quality of life of adolescents and young adults with CHD. The other assessed the effects of a 52-week eHealth intervention on physical fitness and health-related quality of life in adolescents with CHD [27,31].

Web-based portals are digital platforms that enhance patient education, engagement, and communication. They provide accessible information about CHD, treatment options, and self-management strategies. These portals also facilitate communication between patients, families, and health care providers, ensuring continuous support and information exchange. Only 1 study assessed the effectiveness of these interventions in improving adolescent patient knowledge and involvement and supporting physicians in communicating with their patients [29]. Videotape presentations prepare children and their families for medical procedures and hospital stays. The videos use engaging, playful, and child-friendly formats, such as fictional or animated characters and stories, to simplify complex medical concepts and make them less intimidating. In 1982, a study evaluated the impact of a 16-minute videotape presentation on children’s knowledge and coping skills during cardiac catheterization. The study featured a fictional lion who presented the videotape to guide hospitalized children through the events, sights, sounds, and sensations associated with the cardiac catheterization procedure [35].

Of the 11 studies reviewed, 7 offered disease education using the following methods: 3D patient-specific models, empowerment-based health education programs, web-based portals, and videotape presentations [28,29,33-37]. Four studies focused on improving self-management skills using 2 strategies: habit formation interventions and rehabilitation interventions [27,30-32]. Five studies aimed to teach coping strategies using 3 approaches: empowerment-based health education programs, rehabilitation interventions, and videotape presentations [27,31,34-36] (Figure 5). It is important to note that some studies addressed more than 1 aspect, which is why the numbers overlap across different categories.

#### Intervention Duration

The duration of educational interventions varied across the studies, as shown in Figure 5. Some interventions were brief sessions within routine visits, while others lasted up to 1.5 years. Follow-ups also differed. Some interventions had no follow-ups, while others included assessments at intervals of 1 month, 6 months, 1 year, or 2 years.

In total, 2 of 3 studies using 3D patient-specific models were integrated into routine outpatient visits without a specified duration. Another study used digital 3D heart models during a 30-minute tele-education session instead of in person. After the session, patients received a USB drive containing a video of their 3D heart and digital files for potential self-learning, with no follow-up mentioned [28,33,37]. In contrast, habit formation interventions, such as the oral health promotion intervention and a multifaceted physical activity intervention, spanned extended periods. Oral health promotion intervention lasted 1.5 years with sessions at baseline, 6, 12, and 18 months, followed by a 24-month follow-up. The multifaceted physical activity intervention spanned 6 months, including a 3-day workshop and monthly conference calls, with a follow-up period of 6 months [30,32].

As part of the empowerment-based health education program, 1 study included a 40-minute face-to-face education session with individualized instructions on the second day after surgery. There were also 2 monthly 10-minute telephone calls after the discharge to discuss the child’s care and modify action plans. Subsequent assessments were conducted at 1 month and 3 months after surgery. Another study, the Congenital Heart Disease Intervention Program-Family intervention, involved a 1-day workshop and a 6-month follow-up session and assessment [34,36]. Using the rehabilitation interventions strategy, 1 study implemented the QUALI-REHAB cardiac rehabilitation program, consisting of a 12-week program with 5 days of hospitalization at the rehabilitation center and home-based training. Recall sessions were held every 3 weeks at the center, with a final evaluation at the end of week 12 and a 12-month follow-up period for outcome measurements. Another program, the Paediatric Rehabilitation for Vanguard in Lifeskills (PReVaiL), lasted 52 weeks, with a follow-up assessment conducted after 1 year. Patients received group-based health education sessions lasting 45 minutes and individual counseling sessions lasting 15 minutes [27,31].

Additionally, 1 study introduced a web-based portal during outpatient visits that could be used anytime, with follow-up assessments conducted 1 month after the visit [29]. Finally, 1 study conducted a 16-minute videotape presentation session 1 day before procedures, with a follow-up undertaken 4 to 6 weeks after discharge from the hospital [35]. In total, 3 studies had a 1-month follow-up timeline, while 2 had 6 months and 2 had 1 year.

### Study Design and Stakeholder Involvement

#### Overview

The second emergent theme is “study design and stakeholder involvement,” with subthemes of “study design” and “target age groups and stakeholder involvement.” Study design focuses on the methodologies used in the studies and the size of participant samples. Target age groups and stakeholder involvement examines the target population’s age range and impact on stakeholder roles. It analyzes whether parents, caregivers, or health care providers are involved in delivering or evaluating the intervention and identifies the target population. Together, these address KQ2: What are the study designs, target populations, and the roles of various stakeholders in these educational interventions?

#### Study Design

In total, 8 of the 11 studies used an RCT design, while 1 study included a pilot phase followed by an RCT [27-32,34-36]. The sample sizes in these RCTs ranged from 53 to 250, with a mean of 126.11 (SD 43.95). The intervention group size ranged from 31 to 125, with a mean of 63 (SD 25.20), and the control group size ranged from 22 to 125, with a mean of 62.78 (SD 24.60).

These RCTs varied in their approach, including prospective randomized clinical trials; questionnaire-based feasibility and acceptability studies; prospective clinical trials; single-center, single-blinded, randomized controlled trials; prospective, multicenter, randomized, controlled, parallel-arm studies; cluster randomized controlled trials; and multicenter stepped-wedge implementation trials. Additionally, 2 studies adopted observational study designs [33,37]. The observational studies explored a single-center cross-sectional study and a prospective pre-post study, with sample sizes of 46 and 22, respectively.

**Figure 6:**
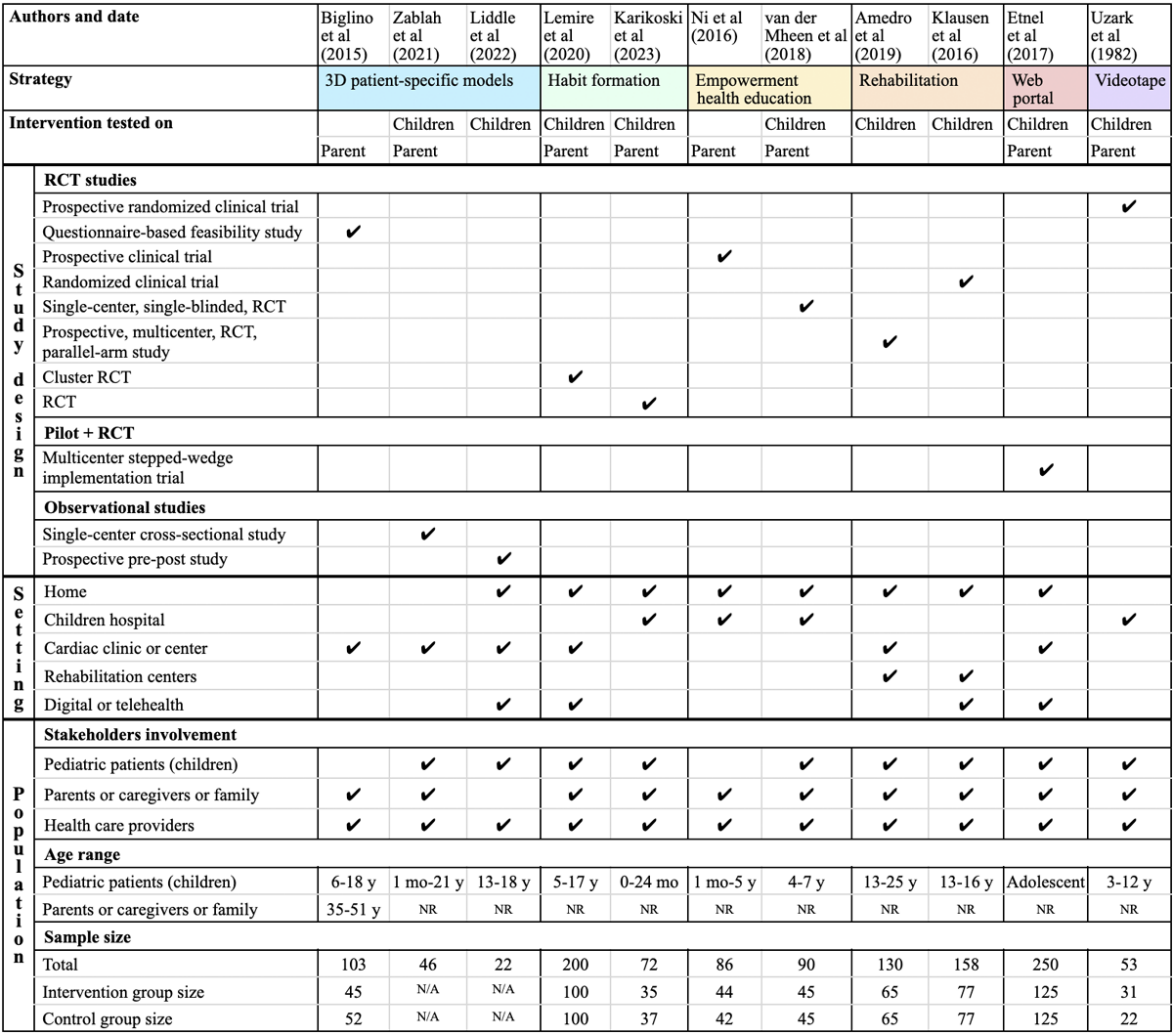
Study design and population characteristics [27-37]. N/A: not applicable; NR: not reported; RCT: randomized controlled trial.

Reviewed studies used diverse settings for their interventions. Three studies implemented interventions across home settings, cardiac clinics, and digital or telehealth platforms [29,32,33]. Another 3 studies implemented interventions at home and children’s hospitals [30,34,36]. One study used interventions in home settings, cardiac clinics, and rehabilitation centers [27]. Additionally, 1 study used home settings, rehabilitation centers, and digital or telehealth platforms [31]. In total, 2 studies were conducted at cardiac centers [28,37], while 1 was conducted exclusively at a hospital [35]. The study designs and settings are summarized in Figure 6.

#### Target Age Groups and Stakeholder Involvement

In the reviewed studies focusing on educational interventions for children with CHD, stakeholder involvement varied depending on the participants’ age range. This variation influenced the roles played by different stakeholders, such as parents or caregivers and health care providers, within these interventions.

The primary emphasis of this review was on educational interventions tailored for children with CHD, although not all studies exclusively targeted children as participants. Several studies also included parents of children with CHD within specific age groups to enhance the overall health outcomes for these children. Among the 11 studies examined, 2 exclusively focused on parents [28,34], while 6 involved both parents and children [29,30,32,35-37]. Additionally, 3 studies specifically targeted children aged 13 to 18, 13 to 25, and 13 to 16 years [27,31,33]. The youngest participants in the studies targeting just children with CHD were aged 13 years.

In a study conducted by Uzark et al [35], the researchers assessed the impact of a videotape presentation on the knowledge and coping of children with CHD aged 3 to 12 years during hospitalization for cardiac catheterization. The study also targeted the parents of these younger children to improve outcomes for both children and parents. Another study by Biglino et al [28] investigated the effectiveness of 3D patient-specific models of CHD as a communication tool during cardiology consultations for pediatric patients aged 6 to 18 years and their parents. The age range of parents included in this study was 35 to 51 years.

Lemire et al [32] studied children with CHD aged 5 to 17 years and their parents. The intervention aimed to assess whether providing resources and protocols would enable clinicians to counsel about physical activity during every pediatric cardiology appointment. In another study, Karikoski et al [30] targeted children with CHD up to 24 months old and their parents to investigate the effectiveness of repeated counseling provided by a dental hygienist in improving oral health behavior during the first 1000 days of life. They aimed to improve parental hygiene habits with the expectation that it would also enhance young children’s hygiene habits.

Ni et al [34] evaluated the effectiveness of an empowerment-based health education program for parents caring for children aged 1 month to 5 years who had undergone corrective surgery for CHD. Their goal was to improve the health outcomes for the children and not the parents. Van der Mheen et al [36] assessed the effects of a program on parental mental health and the psychosocial well-being of children with CHD aged 4 to 7 years. This program also involved siblings to help normalize the child’s CHD position within their family dynamic.

In all 11 studies, key stakeholders—children, parents or caregivers, and health care providers—actively participated in the interventions. In total, 8 studies involved all these stakeholders [27,29-32,35-37], while 2 involved parents and health care providers [28,34], and 1 included children and health care providers [33]. The age ranges in these studies varied widely, from birth to 25 years. However, the age range for parents was not consistently provided across the studies. Only 1 study specified the age range for parents as 35 to 51 years, with children aged 6 to 18 years [28]. Figure 6 provides details of population specification, including stakeholder involvement, age ranges, and sample sizes across the reviewed studies.

### Evaluation Methods and Outcome Objectives

#### Overview

Theme 3, “evaluation methods and outcome objectives,” includes 2 subthemes: “assessed outcome” and “data collection and analysis techniques.” Assessed outcomes discusses the specific objectives and goals the interventions aimed to achieve. Data collection and analysis techniques examines the methods and approaches used to gather and analyze data. Together, these subthemes address KQ3: What outcomes are assessed, and what approaches are used for assessment?

#### Assessed Outcomes

**Figure 7:**
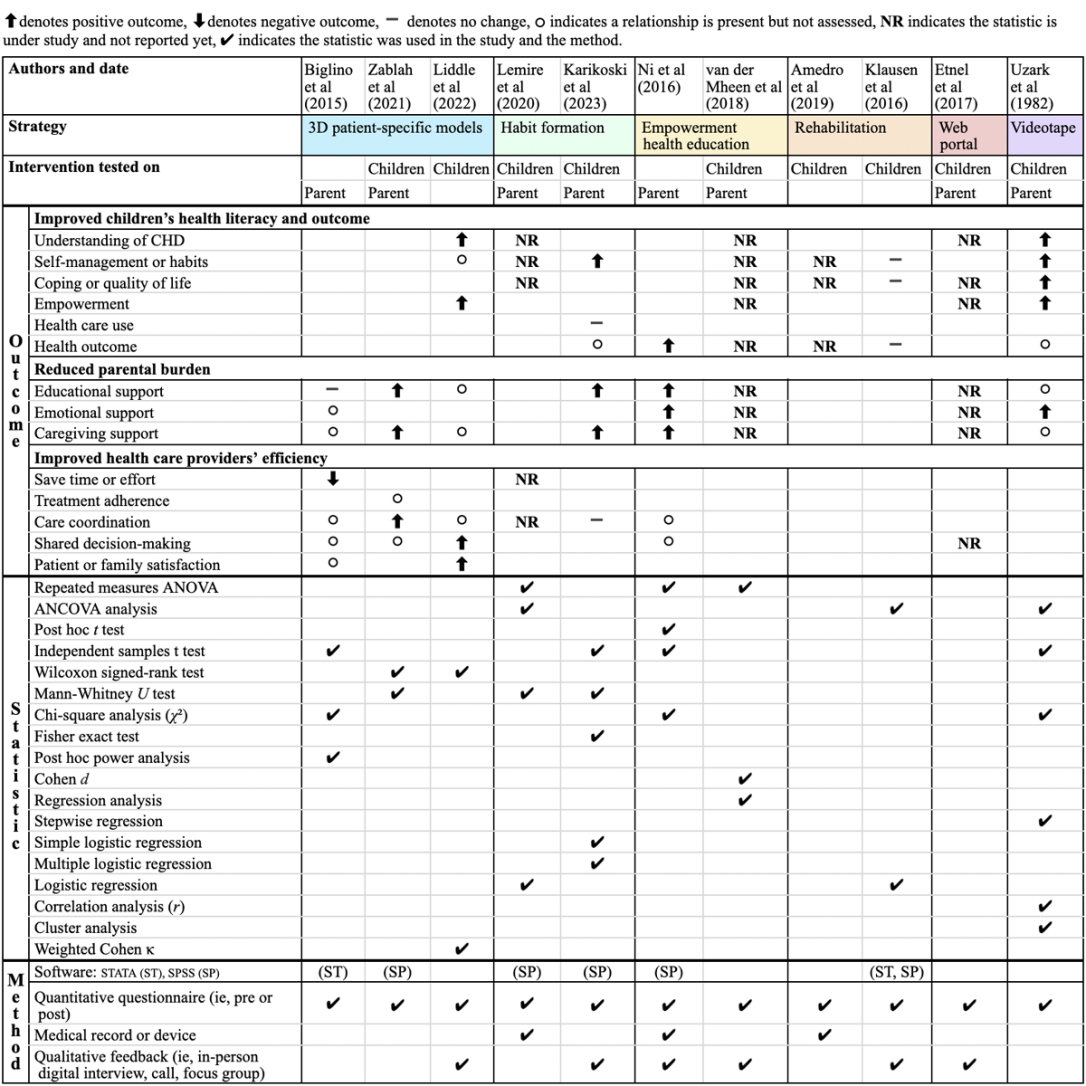
Evaluation methods and outcomes objectives [27-37]. ANCOVA: analysis of covariance; CHD: congenital heart disease.

The outcomes assessed represent the specific objectives the reviewed studies measure to evaluate the effectiveness of their interventions. This scoping review categorized assessed outcomes into three main categories: (1) improved children’s HL and outcome, (2) reduced parental burden, and (3) improved efficiency of health care providers. Figure 7 summarizes these categories, demonstrating the varied impacts of interventions on children with CHD, their parents or caregivers, and health care providers.

Improving children’s HL involves various suboutcomes. These include understanding CHD, self-management and habits, coping and quality of life, empowerment, health care use, and health outcomes. Two studies evaluated the understanding of CHD, and both reported increased knowledge following the intervention [33,35]. Meanwhile, 3 other studies are still under investigation [29,32,36]. Three studies were conducted on self-management and habits. In total, 2 showed positive impacts [30,35], while 1 showed no change or impact [31]. Furthermore, 1 study found a relationship between the intervention and self-management and habits, indicating a need for further investigation in future studies [33]. Ongoing research is being conducted in 3 more studies under investigation [27,32,36].

Two other studies assessed coping and quality of life, one showing no change after intervention [31], and one showing improvement [35], while ongoing investigations continue in 4 studies [27,29,32,36]. Regarding empowerment, 2 studies showed improvement [33,35], with ongoing investigations in 2 more studies [29,36]. Health care use was evaluated once, with no observed difference [30]. Health outcomes were assessed in 2 studies, one indicating improvement [34] and the other showing no change [31]. Additionally, 2 studies without assessments reported a relationship between health outcomes and the intervention [30,35], while ongoing investigations are underway in 2 studies [27,36].

Reducing parental burden focused on various suboutcomes, including educational, emotional, caregiving, and financial support. In total, 4 studies assessed educational support, with 3 showing improvement [30,34,37] and 1 showing no change [28]. Two studies evaluated emotional support, both indicating improvement [34,35]. One study identified a relationship between intervention and emotional support without formal assessments [28], while 2 studies are under investigation [29,36]. Three studies assessed caregiver support, all of which showed improvement [30,34,37]. Similarly, 3 studies identified a relationship that needs further assessment [28,33,35], and 2 are still under investigation [29,36]. Financial support was not assessed in any of the reviewed studies.

Furthermore, regarding enhancing the efficiency of health care providers, one study assessed saving time and effort but found no significant time savings [28], while another study on this topic is still ongoing [32]. Treatment adherence was not directly assessed in any reviewed study, although 1 study noted a relationship without formal assessment [37]. Two studies evaluated care coordination, one indicating improvement [37] and another showing no change [30]. In total, 3 studies identified a relationship between the intervention and care coordination but did not conduct formal assessments [28,33,34], and 1 study is currently under investigation [32]. Shared decision-making and patient or family satisfaction were each assessed in 1 study, both showing improvement [33]. However, another study still investigates shared decision-making [29]. Additionally, 3 studies found a relationship between the intervention and shared decision-making [28,34,37], while 1 reported a relationship with patient or family satisfaction [28]. These findings suggest avenues for future research to assess these relationships more deeply.

#### Data Collection and Analysis Techniques

Across the 11 reviewed studies, various assessment methods and statistical analyses were used to evaluate the effectiveness of interventions for children with CHD. These methods and analyses provided comprehensive insights into the outcomes measured and the statistical rigor applied.

As a statistical software, SPSS (IBM Corp) was predominantly used in 5 studies [30-32,34,37], reflecting its widespread utility for quantitative data analysis in health care research. Data collection methods varied, with all studies using questionnaires, often administered before and after the intervention. In total, 3 studies relied solely on questionnaires and quantitative methods [28,35,37], while 5 studies integrated questionnaire data with qualitative feedback from in-person or digital interviews, calls, or focus groups [29-31,33,36]. Two studies incorporated medical records or devices alongside questionnaires [27,32], and 1 used all 3 methods, including questionnaires, qualitative feedback, and medical records or devices [34].

Statistical analyses used in the reviewed studies to analyze the data included a wide range of methods. The most frequently used statistical analysis method was the independent samples *t* test, used in 4 studies primarily as pre- and postassessments [28,30,34,35]. Other common analyses included repeated measures ANOVA [32,34,36], analysis of covariance analysis [31,32,35], Mann-Whitney *U* test [30,32,37], and chi-square analysis [28,34,35], each used 3 times to assess various outcomes. Several studies have also applied regression analysis techniques such as stepwise regression [35], simple logistic regression [30], multiple logistic regression [30], and logistic regression [31,32].

Among the 11 studies, 2 statistical approaches were used in 3 studies [31,33,37]. Notably, 1 study in 1982 used 6 statistical techniques, including analysis of covariance analysis, independent samples *t* test, chi-square analysis, stepwise regression, correlation analysis (*r*), and cluster analysis [35]. Figure 7 provides an overview of the statistical approaches used across the studies, highlighting a range from 2 to 6 approaches per study.

The reviewed studies used various methods and statistical techniques, indicating no single approach to achieving their objectives. The frequent use of quantitative methods, primarily involving stakeholders other than children with CHD, suggests the need for tailored methods and techniques for this patient demographic.

## Discussion

### Principal Findings

#### Overview

This scoping review identified 11 studies conducted between 1982 and 2023, focusing on educational interventions for children with CHD. The studies included 9 RCTs and 2 observational studies. These studies used various intervention strategies, durations, study designs, and evaluation methods, offering a comprehensive overview of current research in this field. Six primary types of educational strategies were identified: 3D patient-specific models (n=3), habit formation interventions (n=2), empowerment-based health education programs (n=2), rehabilitation interventions (n=2), web-based portals (n=1), and videotape presentations (n=1). These interventions varied in duration, ranging from brief sessions during outpatient visits to programs lasting up to 1.5 years. Follow-up periods also varied, with 3 interventions having no follow-up, 2 having a 6-month follow-up period, 3 having follow-ups ranging from 4 to 6 weeks, and 1 study having a follow-up period of 24 months. The primary goals of these interventions were to improve the quality of life and coping strategies, self-management skills, and knowledge of children with CHD and their families. Among these studies, 3 interventions specifically targeted children above 13 years of age, 2 focused on parents, and 6 involved both children and parents. The primary statistical method used was the independent samples *t* test, used in 4 studies for pre- and postassessments. Outcome assessments focused on children’s HL, reducing parental burden, and improving the efficiency of health care providers. These findings reveal the potential and the limitations of current health educational interventions, highlighting the need for more child-centric approaches to engage younger patients with CHD.

#### Limited Interventions for Children With CHD

This scoping review highlights the potential of educational interventions to significantly improve chronic care management for children with CHD and their families, aligning with previous findings [10-13,40-45]. Existing research indicates that well-informed pediatric patients can delay or prevent secondary illnesses, enhance their quality of life, and reduce health care costs [46-50]. However, many children with chronic conditions lack sufficient understanding of their illnesses, leading to confusion, anxiety, and other complications [51-54]. One contributing factor is the reliance on parents by health care providers to educate their children, assuming effective transmission of information [55,56]. Additionally, the development of interventions has mostly neglected children’s perspectives, focusing primarily on feedback from health care providers and parents. Younger children face barriers to participation due to limited attention spans, difficulty understanding abstract concepts, and challenges in expressing their needs [57,58]. As a result, interventions typically depend on parental feedback as a proxy for testing and design insights [59].

The review also found that educational materials were predominantly designed for teenagers or parental caregivers, lacking age-appropriate and engaging solutions for children younger than 13 years of age. Among the 11 studies reviewed, only 3 [27,31,33] exclusively targeted children, focusing on those aged 13 years and older, likely due to usability challenges for younger children [58,60-62]. This is the case despite the review’s specific focus on educational interventions for children with CHD. The concept that “children are not small adults” is widely acknowledged in pediatric care. It emphasizes that children represent a unique population with their own culture, norms, and complexities [63-65]. This understanding underscores the necessity for developing more inclusive and age-appropriate educational interventions for younger children with CHD.

#### Limited Engaging Strategies

Among the reviewed studies, 3D modeling emerged as a prominent strategy for educating pediatric patients and their families about CHD [28,33,37]. This approach offers engaging learning and improves communication between families and health care providers. However, despite its interactivity, 3D modeling and the other 5 strategies examined in this review lack engaging, playful interactions. Earlier educational tools, such as videotape presentations featuring friendly and playful characters like a fictional lion, effectively engaged young patients by making complex medical procedures more understandable and less intimidating through storytelling [35].

Playful strategies naturally engage children and facilitate learning through play. By communicating complex health information in an age-appropriate and engaging manner, they make a painful and tedious subject more approachable. In the 1920s, nurses Nightingale and Erikson first recognized the importance of systematizing play sessions to improve children’s hospitalization experience and adherence to medical procedures [66-73]. Since then, strategies like pretend play [74,75] and serious games [76,77] have been used for chronic care management, educating children about their conditions, and helping them manage their fears. However, these strategies are rarely used for children with CHD. The evolution of educational interventions from videotape presentations in 1982 to patient-specific 3D printing indicates significant technological advancements. Integrating engaging and playful approaches into current interventions could substantially boost their effectiveness by involving children directly in their health care journey through their own language: play.

#### Enhancing Methodological Approaches

The majority of the reviewed studies used RCTs [27-32,34-36], reflecting robust methodologies for evaluating educational interventions for children with CHD. However, except for 1 [33], many studies primarily assessed parental outcomes as proxies without adequately evaluating specific outcomes for pediatric patients themselves [28-30,32,34-37]. This highlights a gap in HL measures tailored for children with CHD. While parental involvement is essential, directly assessing children’s HL, coping mechanisms, and overall well-being is equally vital.

One notable strength of these studies was the involvement of various stakeholders in intervention delivery, including pediatric patients, parents or caregivers, and health care providers. Eight studies involved all stakeholders, ensuring a comprehensive approach [27,29-32,35-37]. Nevertheless, there is a need for more extensive engagement, particularly with pediatric patients, throughout the intervention’s ideation, design, and implementation phases. Early involvement of stakeholders, including children as design partners, enhances the integration of interventions into routine care, ensuring practicality, feasibility, and alignment with clinical needs [58,78-82].

While most studies used questionnaires supplemented by qualitative feedback, there is a major focus on quantitative approaches. Incorporating more qualitative studies in the initial stages could help identify challenges, barriers, and desires more effectively. Unlike quantitative methods, qualitative methods aim to explore, narrate, and explain phenomena, making sense of complex realities. Health interventions could develop as an outcome of qualitative research [83,84]. Statistical analyses were diverse, with the independent samples *t* test being the most commonly used method, typically involving defined objectives and pre- and postassessment measures. Despite the rigorous methodologies, there was a lack of long-term longitudinal studies to assess the sustained effectiveness of the interventions [85]. Most studies had follow-up periods ranging from 1 month to 1 year, with only 1 study extending to 24 months [30]. This highlights the need for future research to include extended follow-up periods to understand the long-term impact of educational interventions on children with CHD and their families.

By addressing these methodological gaps and expanding stakeholder engagement, particularly with pediatric patients, we can enhance the effectiveness of health educational interventions. Designers, health care providers, and policymakers should prioritize developing and implementing solutions with all stakeholders, not just for them. This collaborative approach can enhance care quality, coordination, and outcomes for children, families, and health care providers.

One effective strategy is to integrate engaging learning tools—both digital and physical—through play. For example, a playful, educational toy similar to Rufus the Bear with Diabetes (Empath Labs), used in diabetes education, can help children manage their health. A comparable toy featuring a simplified heart model allows children to explore their anatomy and medical routines through hands-on play, making complex concepts more accessible. Designed to meet developmental needs, these tools can reduce anxiety, foster independence, and help children manage fears through pretend play. By prioritizing children’s needs rather than relying on parents to convey information, we ensure that health information remains relatable and engaging. Ultimately, these child-led strategies empower families to build the knowledge and resilience necessary for effectively managing CHD. Such efforts could improve health outcomes during the transition to adulthood, enhance autonomy in managing CHD, and streamline education and health care delivery.

### Limitations

This scoping review has offered valuable insights but has limitations. First, the search was limited to English-language publications, which may have excluded relevant studies published in other languages. Second, despite efforts to conduct a comprehensive search, it is possible that some relevant studies were missed, thus introducing selection bias. Finally, the inclusion criteria were restricted to published peer-reviewed studies, which means that relevant gray literature and unpublished studies were excluded, introducing publication bias. It is essential to consider these limitations while interpreting the findings of this scoping review. Additionally, the limited number of studies meeting the inclusion criteria highlights the scarcity of research that focuses specifically on educational interventions for children with CHD. Moreover, the variability in study designs, intervention types, outcome measures, and follow-up periods across the included studies has limited the ability to conduct a meta-analysis and draw definitive conclusions about the effectiveness of educational interventions for children with CHD.

### Research Directions

Educational interventions have shown promise in enhancing the quality of life, self-management skills, and knowledge of children with CHD and their families. However, there is a pressing need for further research to develop and evaluate HL-focused pediatric care interventions tailored specifically for patients with CHD younger than 13 years of age. Drawing from successful interventions in this review, such as the approach by Uzark et al [35] that engaged both pediatric patients and parents to enhance understanding and coping during hospitalization, offers a promising framework for younger children with CHD. This playful approach significantly improved HL, empowerment, and self-management skills. While this study focused exclusively on children with CHD and health educational interventions, future research could draw insights from playful interventions designed for other pediatric conditions like cystic fibrosis and diabetes. As part of our multiphase research project, this comparative approach will inform the iterative development of our health education intervention for younger children with CHD and their families.

Additionally, since no studies included in this review used qualitative approaches such as co-design, our research would prioritize integrating such methodologies to involve all stakeholders, including children, early on. This would enhance the relevance and effectiveness of the interventions. Acknowledging challenges and working with all stakeholders toward finding solutions is essential, as simply ignoring the problem will not lead to progress. Involving children as design partners, despite all the barriers, can ensure that the interventions are engaging, relevant, and effective in meeting the unique needs of children with CHD.

### Conclusions

Educational interventions promise to enhance the quality of life, self-management skills, and knowledge of children with CHD and their families. However, insufficient evidence to support educational interventions for this pediatric population highlights a significant gap in the literature. While this scoping review aimed to identify these gaps, the scarcity of evidence highlights the need for further research. Advocating for such research is crucial to guide designers, health care providers, and policymakers in delivering effective interventions tailored to the specific needs of children with CHD. There is a clear need for more research explicitly addressing pediatric care interventions for children with CHD, focusing on developing age-appropriate, engaging, and engaging educational interventions. Improving HL in pediatric patients can reduce parental educational burden and increase health care provider efficiency by improving communication and patient empowerment.

## Appendix

Multimedia Appendix 1 PRISMA-ScR (Preferred Reporting Items for Systematic Reviews and Meta-Analyses Extension for Scoping Reviews) checklist.

Multimedia Appendix 2 Search strategy.

Multimedia Appendix 3 Study characteristics.

Multimedia Appendix 4 Excluded full-text studies.

## Abbreviations

CHD: congenital heart disease
HL: health literacy
KQ: key question
PICOTS: population, intervention, comparator, outcome, timing, and setting
PRISMA: Preferred Reporting Items for Systematic Reviews and Meta-Analyses
PRISMA-ScR: Preferred Reporting Items for Systematic Reviews and Meta-Analyses Extension for Scoping Reviews
RCT: randomized controlled trial
