# Supplementary material for: Exploring Health Educational Interventions for Children With Congenital Heart Disease: Scoping Review
Source: JMIR Pediatr Parent. 2025 Jan 24;8:e64814. doi: 10.2196/64814 (PMC11806270; doi:10.2196/64814)
Supplement: Multimedia Appendix 2 [file pediatrics_v8i1e64814_app2.docx]

**Ovid MEDLINE(R) ALL <1946 to March 01, 2024>**

**APA PsycInfo <1987 to February Week 5 2024>**

| **Line Number** | **Search String** | **Number of Articles** |
| --- | --- | --- |
| 1 | exp Patient Education as Topic/ or health literacy.mp. or exp Health Education/ or exp Health Knowledge, Attitudes, Practice/ or exp Health Literacy/ | 397751 |
| 2 | exp Child, Preschool/ or pediatric.mp. or exp Pediatrics/ or exp Child/ | 2395156 |
| 3 | paediatric.mp. | 84397 |
| 4 | congenital heart disease.mp. or exp Heart Defects, Congenital/ | 185435 |
| 5 | 2 or 3 | 2418856 |
| 6 | 1 and 4 and 5 | 258 |
| 7 | limit 6 to english language | **235** |

**WEB OF SCIENCE**

4: TS=(“Health Literacy” OR “Healthcare Literacy” OR “Medical Literacy” OR “Health Understanding” OR “Health Education” OR “Healthcare Education” OR “Health Information Literacy” OR “Medical Comprehension” OR “Healthcare Knowledge” OR “Health Knowledge” OR “Health Proficiency” OR “Health Awareness” OR “Medical Awareness” OR “Health Competency” OR “Health Communication” OR “Information Literacy” OR “Patient Education” OR “Health Promotion” OR “Health Teaching”) AND TS=(Children OR Child* OR Kid OR Kids OR Girl* OR Boy OR Boys* OR Toddler* OR Childhood OR Preschool* OR Pre-school* OR Kindergarten* OR School OR Minors OR Pediatric* OR Paediatric*) AND TS=(“Congenital Heart Defects” OR “Child Heart Disease” OR “Heart Defects” OR “Congenital Heart Disease” OR “Pediatric Cardiology” OR “Paediatric Cardiology” OR “Cardiac Defect*”) and English (Languages) and NOT Document Type: Editorial Materials or Meeting Abstarct

**Results: 102**

**ACM Digital Library**

[[All: "health literacy"] OR [All: "healthcare literacy"] OR [All: "medical literacy"] OR [All: "health understanding"] OR [All: "health education"] OR [All: "healthcare education"] OR [All: "health information literacy"] OR [All: "medical comprehension"] OR [All: "healthcare knowledge"] OR [All: "health knowledge"] OR [All: "health proficiency"] OR [All: "health awareness"] OR [All: "medical awareness"] OR [All: "health competency"] OR [All: "health communication"] OR [All: "information literacy"] OR [All: "patient education"] OR [All: "health promotion"] OR [All: "health teaching"]] AND [[All: children] OR [All: child*] OR [All: kid] OR [All: kids] OR [All: girl*] OR [All: boy] OR [All: boys*] OR [All: toddler*] OR [All: childhood] OR [All: preschool*] OR [All: pre -school*] OR [All: kindergarten*] OR [All: school] OR [All: minors] OR [All: pediatric*] OR [All: paediatric*]] AND [[All: "congenital heart defects"] OR [All: "child heart disease"] OR [All: "heart defects"] OR [All: "congenital heart disease"] OR [All: "pediatric cardiology"] OR [All: "paediatric cardiology"] OR [All: "cardiac defect*"]]

**Results: 25**

**SCOPUS**

TITLE-ABS-KEY ( "Health Literacy" OR "Healthcare Literacy" OR "Medical Literacy" OR "Health Understanding" OR "Health Education" OR "Healthcare Education" OR "Health Information Literacy" OR "Medical Comprehension" OR "Healthcare Knowledge" OR "Health Knowledge" OR "Health Proficiency" OR "Health Awareness" OR "Medical Awareness" OR "Health Competency" OR "Health Communication" OR "Information Literacy" OR "Patient Education" OR "Health Promotion" OR "Health Teaching" ) AND TITLE-ABS-KEY ( children OR child* OR kid OR kids OR girl* OR boy OR boys* OR toddler* OR childhood OR preschool* OR pre-school* OR kindergarten* OR school OR minors OR pediatric* OR paediatric* ) AND TITLE-ABS-KEY ( "Congenital Heart Defects" OR "Child Heart Disease" OR "Heart Defects" OR "Congenital Heart Disease" OR "Pediatric Cardiology" OR "Paediatric Cardiology" OR "Cardiac Defect*" ) AND ( LIMIT-TO ( LANGUAGE , "English" ) ) AND ( EXCLUDE ( DOCTYPE , "ed" ) OR EXCLUDE ( DOCTYPE , "no" ) OR EXCLUDE ( DOCTYPE , "sh" ) OR EXCLUDE ( DOCTYPE , "le" ) OR EXCLUDE ( DOCTYPE , "ch" ) OR EXCLUDE ( DOCTYPE , "bk" ) )

**Results: 473**

**EBSCOhost**

AB ( =(“Health Literacy” OR “Healthcare Literacy” OR “Medical Literacy” OR “Health Understanding” OR “Health Education” OR “Healthcare Education” OR “Health Information Literacy” OR “Medical Comprehension” OR “Healthcare Knowledge” OR “Health Knowledge” OR “Health Proficiency” OR “Health Awareness” OR “Medical Awareness” OR “Health Competency” OR “Health Communication” OR “Information Literacy” OR “Patient Education” OR “Health Promotion” OR “Health Teaching”) ) AND AB ( (Children OR Child* OR Kid OR Kids OR Girl* OR Boy OR Boys* OR Toddler* OR Childhood OR Preschool* OR Pre-school* OR Kindergarten* OR School OR Minors OR Pediatric* OR Paediatric*) ) AND AB ( (“Congenital Heart Defects” OR “Child Heart Disease” OR “Heart Defects” OR “Congenital Heart Disease” OR “Pediatric Cardiology” OR “Paediatric Cardiology” OR “Cardiac Defect*”) )

English and Academic Journals and Peer Reviewed 57

**No duplication 26**
