# Supplementary material for: Exploring Health Educational Interventions for Children With Congenital Heart Disease: Scoping Review
Source: JMIR Pediatr Parent. 2025 Jan 24;8:e64814. doi: 10.2196/64814 (PMC11806270; doi:10.2196/64814)
Supplement: Multimedia Appendix 3 [file pediatrics_v8i1e64814_app3.docx]

**Table S1.** Study identification.

| **ID** | **Author Year** | **Country** | **Study Title** | **Study Design** | **Date of Study** | **Setting** | **Objective Purpose** |
| --- | --- | --- | --- | --- | --- | --- | --- |
| 1 | Ni et al. (2016)  [36] | China | An empowerment health education program for children undergoing surgery for congenital heart diseases | Prospective Clinical Trial | Nov. 2010 -  Nov. 2011 | Children’s Hospital of Soochow University, Suzhou City, China. | To evaluate the effectiveness of an empowerment health education program for improving caregiving knowledge, caring behaviors, and self-efficacy of parents caring for children after corrective surgery for CHD |
| 2 | Biglino et al. (2015)  [30] | England | 3D-manufactured patient-specific models of congenital heart defects for communication in clinical practice: feasibility and acceptability | Questionnaire-Base  d. (randomization) - Feasibility and acceptability study | NR | Outpatient clinic, cardiology follow-up visits | To assess the communication potential of 3D patient-specific models of congenital heart defects and their acceptability in clinical practice for cardiology consultations. |
| 3 | Amedro et al. (2019)  [29] | France | Impact of a centre and home-based cardiac rehabilitation program on the quality of life of teenagers and young adults with congenital heart disease: The QUALI-REHAB study rationale, design, and methods | RCT: Prospective, Multicenter, Randomized, Controlled, Parallel Arm Study | NR | The study included 16 CHD centers and 7 cardiac rehabilitation centers in France. A total of 33 investigators were in charge of patient recruitment throughout the country. | The study aimed to assess the impact of a combined center- and home-based cardiac rehabilitation program on the quality of life of adolescents and young adults with congenital heart disease. |
| 4 | van der Mheen et al. (2018)  [38] | Netherland s | The CHIP-Family study to improve the psychosocial wellbeing of young children with congenital heart disease and their families: design of a randomized controlled trial | RCT: Single-Center, Single-Blinded Randomized Controlled Trial | Sep. 2016 -  Sep. 2017 | Department of Pediatric Cardiology, Erasmus Medical Center – Sophia Children’s Hospital, Dutch Patient Association for Congenital Heart Disease | The CHIP-Family study aims to (1) test the effects of CHIP-Family on parental mental health and psychosocial wellbeing of CHD-children and to (2) identify baseline psychosocial and medical predictors for the effectiveness of CHIP-Family. |
| 5 | Uzark et al. (1982)  [37] | USA | Use of videotape in the preparation of children for cardiac catheterization | Prospective Randomized Clinical Trial | Sep. 1979 -  Sep. 1980 | C.S. Mon Children's Hospital, Ann Arbor, Michigan | The purpose of this prospective study was to evaluate the effect of the videotape on (1) the child's knowledge of the events and sensations experienced during hospitalization for cardiac catheterization and (2) the child's coping as evidenced by behavior during cardiac catheterization and adjustment after hospitalization. |
| 6 | Zablah et al. (2021)  [39] | USA | Cardiac catheterization laboratory and the role in effective patient education: A model approach | Single-Center Cross-Sectional Study | Sep. 2019 -  Aug. 2020 | Children's Hospital Colorado | This study reports on the experience using three-dimensional printed models (3DPM) for patient education and compares it with other methods for patients undergoing cardiac catheterizations. The goal is to improve families' and patients' understanding of cardiac anatomy, increase patient satisfaction, and optimize doctor-patient communication. |
| 7 | Liddle et al. (2022)  [35] | USA | Combining patient-specific, digital 3D models with tele-education for adolescents with CHD | Prospective pre-post study | NR | Tertiary pediatric care center | Investigate the feasibility of using digital 3D models with tele-education for adolescents with CHD |
| 8 | Etnel et al. (2017)  [31] | Netherland s | Development of an Online, Evidence-Based Patient Information Portal for Congenital Heart Disease: A Pilot Study | Pilot Study (multicenter stepped-wedge implementation trial) | NR | Four large congenital cardiac centers in the Netherlands (departments of both adult and pediatric cardiology at each of the four participating centers) | The objective of this pilot project was to develop an online information portal that aims to (1) improve patient knowledge and involvement and to subsequently reduce anxiety, depression, and decisional conflict and improve mental quality of life and (2) to support physicians in informing and communicating with their patients. |

| 9 | Karikoski et al. (2023)  [32] | Finland | Early Counseling to Improve Oral Health Behavior in Children with Major Congenital Heart Defects: A Randomized Controlled Trial | Randomized Controlled Trial (National Population-Based) | NR | Children’s Hospital, Helsinki University Hospital, Finland | To investigate the effectiveness of repeat counseling provided by a dental hygienist in improving oral health behavior in children with major CHD during the first 1,000 days of life. |
| --- | --- | --- | --- | --- | --- | --- | --- |
| 10 | Klausen et al. (2016)  [33] | Denmark | Effects of eHealth physical activity encouragement in adolescents with complex congenital heart disease: The PReVaiL randomized clinical trial | Randomized Clinical Trial | 2010 - 2014 | Institute of Sports Medicine in Copenhagen by a team consisting of 3 nurses, 1 health coach specializing in adolescence, and 2 exercise physiologists. The team was blinded to the group allocation of patients. | Evaluate the effectiveness of a 52-week eHealth intervention, comprising the Internet, mobile application, and SMS-based components, on physical fitness, physical activity, and health-related quality of life in adolescents with congenital heart disease (CHD). |
| 11 | Lemire et al. (2020)  [34] | Canada | Impacting child health outcomes in congenital heart disease: Cluster randomized controlled trial protocol of in-clinic physical activity counseling | Cluster Randomized Controlled Trial | NR | Pediatric cardiology clinics in small scale (London, ON), medium scale (Ottawa, ON), and large scale (Edmonton, AB). | The goal is to determine whether providing resources and protocols enables clinicians to counsel about physical activity as part of every pediatric cardiology appointment. Evaluations of healthcare system impact and intervention delivery in small, medium, and large clinics will assess the applicability of this approach to all pediatric cardiac clinics. |

**Table S2.** Participants details.

| **ID** | **Author Year** | **Target Population** | **Intervention Tested on** | **Sample Size** | **Demographic**  (Parents or/and Children) | **Inclusion Criteria**  (Parents / Children) | **Exclusion Criteria**  (Parents / Children) | **CHD**  **Severity Pre and**  **Post-Surgery** |
| --- | --- | --- | --- | --- | --- | --- | --- | --- |
| 1 | Ni et al. (2016)  [36] | Children undergoing surgery for CHD | Parents caring for children after surgery for CHD | n=86  Intervention (n=44) Control (n=42) | C:  1 month to 5 years | P: Children undergoing first-time corrective surgery for CHD,  C: Willingness to participate, Parents signed informed consent | P: Chromosomal defect, recent birth complication,  C: Mental impairment, single-parent caregivers, caregiver but not parent | Pre and  Post-Surgery |
| 2 | Biglino et al. (2015)  [30] | Parents of children with congenital heart disease | Parents of children with congenital heart disease | n=103 Model (n=45)  Control (n=52) | C:   - Mean patient age:   Model: 14±5, Control: 10±6 P:   - Mean parental age:   Model: 44±7, Control: 41±9   - Sex:   Model: (F/M) 33/9, Control: (F/M) 40/12   - Level of Education:   6th form: Model: 13, Control: 15 After 6th form: Model: 7, Control: 7  University graduate: Model: 9, Control: 11 University postgraduate: group: 4, Control: 8  Other: Model: 9, Control: 10 | P: Parents of CHD children. C: CHD Children with recent cardiac MRI. | P: Unwilling/unable parents. C: Patients without recent cardiac MRI, MRI contraindications, Severe cognitive impairments, Other serious medical conditions | NR |
| 3 | Amedro et al. (2019)  [29] | Adolescents and Young Adults with CHD | Patients with a CHD, aged from 13 to 25 years | n=130  Intervention (n=65) Control (n=65) | C:  Age Range: 13 to 25 years old | C: Male/female (13 to 25), CHD patients, defined by the international ACC-CHD classification, CPET performed within the last 3 months with VO2max b 80% of predicted VO2max or VAT b55% of predicted VO2max, Written informed consent for adult patients or legal guardians for teenagers and formal assent for teenagers | C: Various conditions such as contraindications for exercise testing, planned cardiac surgery during the study, recent cardiac events, severe cardiac conditions, pregnancy, musculoskeletal abnormalities, and cognitive impairments | NR |
| 4 | van der Mheen et al. (2018)  [38] | Children with CHD and their Families | Parents and Children | n=90  Intervention (n=45) Control (n=45) | C:  Age Range: 4 to 7 years old | C: Underwent at least one invasive procedure (catheter intervention or surgery) for CHD and are starting or attending kindergarten or primary school (first or second year) at the time of first assessment (as the children are approximately 4–7 years old) | C: Child’s intellectual impairment (IQ < 70) as ascertained by previous standardized assessment or diagnosed by a clinician, Insufficient mastery of the Dutch language, and Prematurely born children (gestational age at birth < 37 weeks) with no other CHD than a patent ductus arteriosus. | - Limited to no residual heart defects after medical intervention - Moderate to severe residual heart defects after medical intervention |

| **ID** | **Author Year** | **Target Population** | **Intervention Tested on** | **Sample Size** | **Demographic**  (Parents or/and Children) | **Inclusion Criteria**  (Parents / Children) | **Exclusion Criteria**  (Parents / Children) | **CHD**  **Severity Pre and**  **Post-Surgery** |
| --- | --- | --- | --- | --- | --- | --- | --- | --- |
| 5 | Uzark et al. (1982)  [37] | Children with congenital heart disease (CHD) and  their parents | Parent and/or Child who were hospitalized for cardiac catheterization | n=53  Experimental (n=31) Control (n=22) | C:   - Age Range: 3 to 12 years old - Mean Age:   Experimental 7.9 (range: 3.6 to 12.4 yrs)  and Control 7.5 (range: 3.6 to 12.9 yrs)   - Sex:   25 girls and 28 boys - Experimental 47% Female and Control 53% Female | C: Children with congenital heart disease (CHD) | C: Acute illness, obvious neurological impairment, or hearing loss | Pre and  Post-Surgery |
| 6 | Zablah et al. (2021)  [39] | Patients, including children undergoing cardiac catheterizations with Parents | Most parents (95.7%) and only the patient (4.3%) answered in two cases. | n=46  Parents (n=44)  Patients (n=2) | C:   - Age: Median 2 yrs (Range: 1 month to 21 years) - Sex: Male 26 (56.5%), Female 20   (43.5%)  - Weight, Kg: 12.1 (3.6–95.8)  - Height, cm: 90.8 (51–191.2) | NR | NR | Pre and  Post-Cardiac Catheterizatio n Procedure |
| 7 | Liddle et al. (2022)  [35] | Adolescents with CHD | Adolescents with CHD | n=22 | C:   - Age: Mean age: 16 years 13 to 18 years   13-15 years: 6 (27.2)  15-18 years:16 (72.8)   - Sex   Male: 10 (45.5), Female: 12 (54.5)   - Race   Caucasian: 15 (68.2), Black: 5 (22.7)  Other: 2 (9.1) | C: - Patients aged 13-18 years, - History of CHD, - Previous cardiac MRI imaging | C: - Moderate/severe developmental delay, - Specific genetic syndromes, -  Non-English speaking, - Other comorbidities | NR |
| 8 | Etnel et al. (2017)  [31] | Teenagers, adult patients, caregivers, or parents of pediatric patients with congenital aortic and/or pulmonary valve disease and/or Tetralogy of Fallot. | Teenagers, adult patients, and caregivers or parents. | - **Phase 1:** Evaluation of the current state of patient Information: Patients   (n = 63), caregivers of pediatric patients (n = 10), physicians (n = 32)   - **Phase 2:** Development of information portal:   adult patients (n = 2), caregivers (n = 2), physicians (n = 6; 2  pediatric cardiologists, 2 adult congenital cardiologists, and 2 congenital cardiac surgeons), and clinical psychologists (n = 2)   - **Last Phses:**   n=250,  (n=125) control and (n=125) intervention | Teenagers, Adults, or Parents/Caregivers | Patients and caregivers visiting the outpatient clinic at the participating centers | NR | NR |

| **ID** | **Author Year** | **Target Population** | **Intervention Tested on** | **Sample Size** | **Demographic**  (Parents or/and Children) | **Inclusion Criteria**  (Parents / Children) | **Exclusion Criteria**  (Parents / Children) | **CHD**  **Severity Pre and**  **Post-Surgery** |
| --- | --- | --- | --- | --- | --- | --- | --- | --- |
| 9 | Karikosk i et al. (2023)  [32] | Families with CHD children born in Finland between April 1,  2017, and  October 31, 2020 | CHD children and Parents | n=72  (CHD-I) intevention n=35 (CHD-C) control n=37 | C:  Age: range: 0–11 months   - less than 12 months - 6 to 12 to 24 months | C: - Children born in Finland April 1, 2017–Oct. 31, 2020, aged less than 12 months, with (a) major CHD potentially included in the criteria of endocarditis prophylaxis or (b) any CHD with surgical repair combined with a chromosomal syndrome (potentially included in the criteria of endocarditis prophylaxis)   - Cardiomyopathy (potential cardiac transplantation recipient) - Chromosomal abnormality or syndrome and any invasive intervention (surgery/cath) for CHD or likely to require invasive intervention for CHD | C: Neither parent is able to comprehend intervention instructions provided in Finnish   - Child in out-of-home care (e.g., foster care) - Not born in Finland between April 1, 2017, and October 31,   2020 | NR |
| 10 | Klausen et al. (2016)  [33] | Adolescents aged 13–16 years with no physical activity restrictions after repaired complex congenital heart disease. | Adolescents aged 13–16 | n=158  Allocated to SMS text messages (n=81)   - Received allocated intervention (n=57) - Did not receive the allocated intervention (n= 24): did not use the application for at least 2 consecutive weeks during the study period   Allocated to a control intervention (n=77)   - Received allocated intervention (n= 77) - Did not receive the allocated intervention (n=0) | C: Aged 13–16 | C: Age between 13 and 16 years, previous repair for a complex CHD, and assignment to lifelong medical follow-up - No limitations to physical activity - Stable clinical condition - Able to understand Danish language - Willing to participate in the 52-week intervention | C: Residual defects significant for physical activity restrictions, assessed by the participants' regular cardiologist. - Patient health records were manually checked for clinically important co-morbidities that could lead to exclusion. Inability to understand the Danish language  - Unstable clinical condition - Inability to participate in the 52-week intervention due to physical or mental limitations - Refusal to participate | Complex |
| 11 | Lemire et al. (2020)  [34] | Children with CHD | Children with CHD and their Parents | n=200  Intervention (n=100) Control (n=100) | C: Age: 5-17 years | C: Diagnosed with moderate to severe CHD - Age 5-17 years | C: Recent cardiac intervention (catheterization or surgery) within the past 6 months - Syndromes or diagnoses affecting physical activity or ability to complete assessment questionnaires | Moderate to Severe |

**Table S3.** Intervention specification.

| **I D** | **Author Year** | **Intervention**  Name/Picture | **Format** | **Description** | **Comparison** | **Stakeholders' Roles** | **Duration** | **Follow-up** |
| --- | --- | --- | --- | --- | --- | --- | --- | --- |
| 1 | Ni et | Empowerment- | - Face-to-Face | -Standard discharge instructions. - Participating | Standard discharge | **Parent:** | - Initial session: Up to 40 mins | - 1 month |
|  | al. | Based health | Education | in a five-unit program based on empowerment | instructions covering | - Attend education sessions. | - Follow-up calls: Three 10-min | (after |
|  | (2016) | education | Sessions. | education principles: Identification, relationship | 7 content areas for | - Assess caregiving knowledge and | sessions | surgery) |
|  | [36] | program | - Follow-Up | establishment, goal setting, formulation of | caring for the child | self-efficacy. |  | - 3 months |
|  |  |  | Telephone | action plans, dynamic evaluation, and | after surgery, | - Set caregiving goals collaboratively. |  | (after |
|  |  |  | Calls | modification. - Initial face-to-face education | delivered in a | - Develop personalized action plans. |  | surgery) |
|  |  |  |  | session (40 minutes) on the second day after | 20-minute session | - Review and adjust plans regularly. |  |  |
|  |  |  |  | surgery, with individualized instructions. - | using booklets and | - Implement strategies at home. |  |  |
|  |  |  |  | Three monthly 10-minute telephone calls | reading materials. | - Communicate concerns during |  |  |
|  |  |  |  | post-discharge discussing the child's care and |  | follow-up calls. |  |  |
|  |  |  |  | modifying action plans. |  | - Adapt improvement plans. |  |  |
| 2 | Biglino | 3D | 3D Medical | Based on cardiac MRI examinations, | Routine visits without | **Parent:** | Outpatient follow-up routine time | NR |
|  | et al. | Patient-Specific | Model | patient-specific 3D models of congenital heart | the use of 3D models | - Participated in the study | + 5 min questionnaires before and |  |
|  | (2015) | Models of |  | defects were used as visual aids during |  | - Provided feedback | 5 min after the visit |  |
|  | [30] | Congenital |  | cardiology consultations. |  | - Being end users of information |  |  |
|  |  | Heart Defects |  |  |  | **Cardiologists (HCPs):** |  |  |
|  |  | 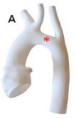 |  |  |  | - Interacted with parents - Explained the child's condition - Assessed parental understanding - Made treatment decisions |  |  |
| 3 | Amedr | The | Combined | - A 12-week program, including both | During the 12-month | **Parent:** | 12 weeks Education | 12-month |
|  | o et al. | QUALI-REHA | Center-based | center-based and home-based training. - | study period, they will | - Parents or legal guardians provided | Totall Study 36 months | follow-up |
|  | (2019) | B Cardiac | and | Hospitalization in the rehabilitation center for | have a regular, | informed consent for minors. |  | period for |
|  | [29] | Rehabilitation | Home-based | the first 5 days, with personalized education, | non-modified | **Children:** Participated |  | outcome |
|  |  | Program | training - | exercise training, and physical therapy sessions. | follow-up with no | **Healthcare Providers:** |  | measurement |
|  |  |  | (Personalized | - From week 2 to 12, participants continue the | rehabilitation | - Pediatric Cardiologists, Adult |  | s |
|  |  |  | Education, | training at home with two individual exercise | program. - Once the | Congenital Cardiologists, Physical |  |  |
|  |  |  | Clinical | sessions/week using a stationary bicycle, | 12-month study | Education Teachers, Psychologists, |  |  |
|  |  |  | Examination, | supervised by a physical education teacher. - | period is over, they | Dieticians, Specialist Nurses, |  |  |
|  |  |  | Cardiopulmona | Three recall sessions are held at the | can participate in the | Physiotherapists, Research Nurses, and |  |  |
|  |  |  | ry Exercise | rehabilitation center every three weeks, | cardiac rehabilitation | Clinical Research Assistants for Study |  |  |
|  |  |  | Testing, | including interval training exercises, reinforced | program. | Conduct and Patient Follow-up. |  |  |
|  |  |  | Supervised | education support, and physical therapy. The |  | * Multicenter involvement with |  |  |
|  |  |  | Interval | program concludes with a final evaluation at |  | harmonization meetings. |  |  |
|  |  |  | Training | the end of week 12. |  | * Nation-wide health-provider company |  |  |
|  |  |  | Exercises) |  |  | for a home-based program delivery. |  |  |
| 4 | van der | CHIP-Family | Workshop | - Separate one-day workshop for parents and | Care as usual (CAU; | **Parents:** | One-day workshop for parents and | Follow-up |
|  | Mheen | intervention | (parent module | children. - Child workshop includes | regular medical | - Participated in the study | children, with a follow-up session | assessment |
|  | et al. |  | and a child | psychological exercises based on cognitive | treatment, no | - Provided feedback | approximately 4 weeks later. | (T2) takes |
|  | (2018) |  | module with | behavioral therapy and sports exercises. Each | psychosocial | **Sibling /Friend:** |  | place 6 |
|  | [38] |  | separate | child can bring a 4- to 10-year-old | intervention) | - Participated to normalize and to |  | months after |
|  |  |  | workshops for | sibling/friend to the workshop. Siblings are |  | stimulate practice at home |  | the initial |
|  |  |  | parents and | involved in the activities, and attention is given |  | **Healthcare Providers:** |  | assessment |
|  |  |  | children) | to them by the hospital staff, aiming to |  | - Provided the intervention, including |  | (T1) |

| **I D** | **Author Year** | **Intervention**  Name/Picture | **Format** | **Description** | **Comparison** | **Stakeholders' Roles** | **Duration** | **Follow-up** |
| --- | --- | --- | --- | --- | --- | --- | --- | --- |
|  |  |  |  | normalize the position of the child with CHD within their family dynamic. - Parent workshop based on the evidence-based CHIP-School focuses on problem prevention therapy, psychoeducation, parenting skills, and medical issues. - Follow-up booster session for parents. |  | Psychologists, Pediatric Cardiologist, Physiotherapists, Psychologists |  |  |
| 5 | Uzark et al. (1982)  [37] | Leonard Z. Lion Presents... Learning About Your Heart Catheterization  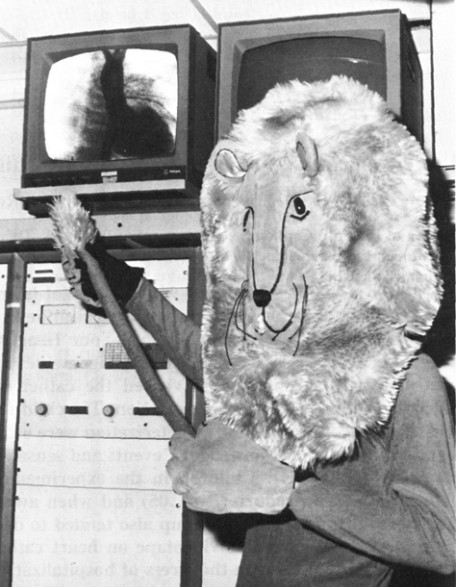 | Videotape:  16-min video presentation developed by the University of Michigan features a friendly fictional lion who appears during a  7-year-old girl's hospitalization for cardiac catheterization to explain the events that take place and to prepare her for the sights, sounds, and sensations to be experienced. | - A day before Parents/children completed the Johnson-Radloff and Helmreich Mood Adjective Checklist (MACL) assessing mood. - Knowledge test administered to each child with ten questions about hospitalization. - The experimental group watched "Leonard Z. Lion Presents... Learning About Your Heart Catheterization." A 16-minute video featuring a friendly lion explains cardiac catheterization preparations. - Mood measured post-video, with knowledge test repeated later. - 2 technicians rated the child's catheterization behavior for nine behaviors. - Parents completed the post-discharge questionnaire from Vernon and Schubman's questionnaire. - Grouped data compared using analysis of covariance. Mood indices derived from MACL. | Watched another unrelated videotape titled "Lion Visits the Dentist." | **Parents/Children:**   - Completed mood checklist and knowledge test, watched the video, and provided feedback. - Parents also completed the post-discharge questionnaire. **Technicians/ Providers:** - Rated child's behavior during catheterization.   **Researchers:**   - Designed, conducted, and analyzed the study. | 16-minute video watching | 4 to 6 weeks after discharge from the hospital. |
| 6 | Zablah et al. (2021)  [39] | NR  Three-Dimensio nal Printed Models (3DPM) | 3DP Medical Model (3DPM) | 3DPMs were created from 3D Rotational Angiography (3DRA) images used during cardiac catheterization procedures. - The models were used in personalized educational sessions with medical professionals before the cardiac catheterization procedure. - These sessions, conducted pre-procedure, provided patients/families with an opportunity to visually explore/understand the child's cardiac anatomy and treatment plan. - Along with interactive discussions facilitated by the 3DPMs and accompanying diagrams or images, complex anatomical structures and treatment options were clarified. - Models used to educate patients/families about the child's cardiac anatomy and treatment plan, empowering them with essential knowledge prior to the procedure. | Traditional methods of patient education, such as videos, drawings, and educational materials. | **Medical Professionals:** (Interventional Cardiologists, Healthcare Providers):   - Conducted personalized educational sessions with patients/their families. - Explained the child's cardiac anatomy and treatment using 3DPMs. - Facilitated interactive discussions, allowing patients/families to explore and understand complex anatomical structures and treatment visually.   **Patients and their Families:**   - Participated in one-on-one educational sessions with medical professionals. - Engaged in discussions about the child's cardiac condition and treatment plan. - Explored and manipulated the 3DPMs to enhance understanding. - Asked questions, sought clarification | NR | NR |

| **I D** | **Author Year** | **Intervention**  Name/Picture | **Format** | **Description** | **Comparison** | **Stakeholders' Roles** | **Duration** | **Follow-up** |
| --- | --- | --- | --- | --- | --- | --- | --- | --- |
| 7 | Liddle et al. (2022)  [35] | NR  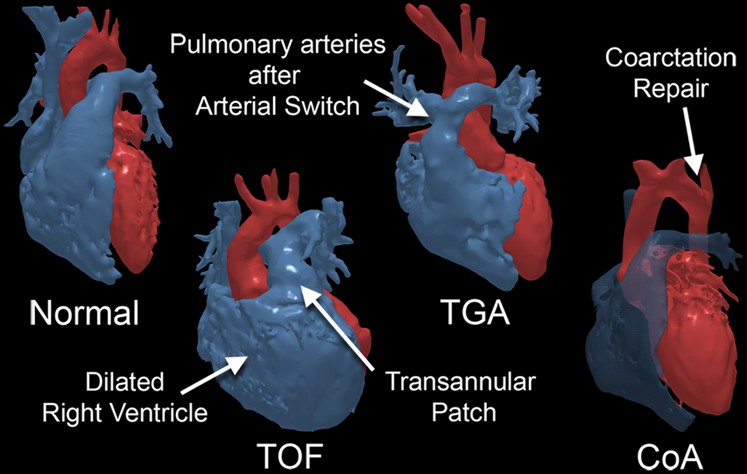 | Digital 3D Heart Models | During tele-education sessions, patient-specific digital 3D heart models created from MRI imaging were utilized. These models provided interactive visualizations of the patient's cardiac anatomy and surgeries.  - Educational sessions conducted via Zoom with a pediatric cardiologist, using Cardiac Review 3D software for digital 3D heart model display. The same cardiologist led all sessions. A standardized curriculum covered normal and patient-specific cardiac anatomy, previous surgeries, transitioning to adult care, and patient wellness. No clinical recommendations were given. Following sessions, patients received a USB drive with a video of their 3D heart and digital files for potential 3D printing. | NR | **Pediatric cardiologists:** Led educational sessions and provided medical expertise. **Patients:** Engaged in sessions and received information about their CHD. **Parents (optional):** Could participate in the sessions.  **Researchers:** Designed and conducted the study and analyzed data. | 30-minute web conference using 3D models. | NR |
| 8 | Etnel et al. (2017)  [31] | NR  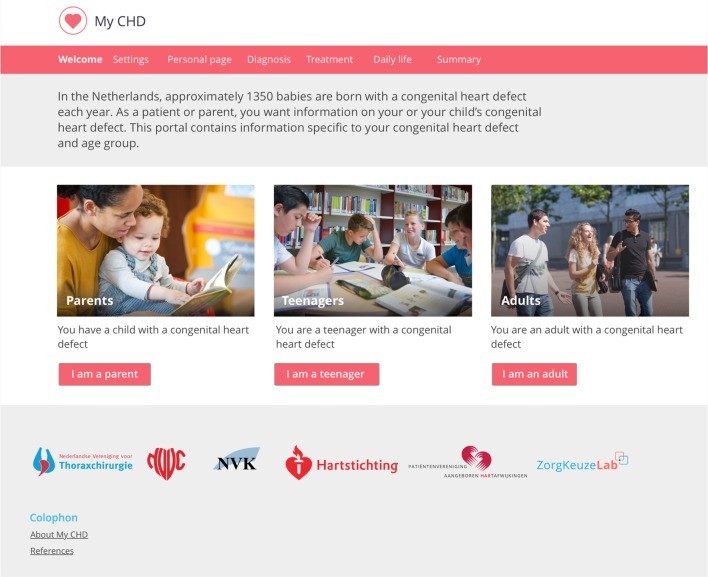 | Web-based Portal  -  An online, evidence-based information portal that is supported by both patients and physicians.  It includes information on diagnosis, treatment, prognosis, psychosocial aspects, and implications for daily life. | Comprehensive online platform providing evidence-based disease- and age-specific medical and psychosocial information about congenital heart disease, including diagnosis, treatment, prognosis, and impact on daily life.  The focus of this pilot project was to develop a nationwide patient-tailored, evidence-based patient information tool to be incorporated into specialist congenital cardiac care developed by and for patients, caregivers, and physicians based on both patient/caregiver and physician preferences. | Outcomes of patients and/or caregivers who have access to the info portal (intervention) with those who do not have access (control) | **Patients and Caregivers:**   - Access the portal to obtain CHD info. - Provide feedback on the usability, comprehensibility, and effectiveness. - Participate in surveys/interviews to assess info needs and preferences. **Physicians:** - Refer patients/caregivers to the portal during clinical consultations. - Use the portal to aid in explaining disease info to patients/ caregivers. - Provide input on the content/format of the portal.   **Researchers:**   - Assess the current state of patient info and gather insights into patient needs. - Design/conduct the implementation trial to evaluate the impact of the info portal on patient outcomes. - Analyze collected data to assess the portal's effectiveness.   **Multidisciplinary Group Members:**   - Collaborate in developing the portal. - Provide expertise in cardiology, psychology, epidemiology, web design. - Review/revise the content, format, and usability of the portal. - The portal is developed in collaboration with Dutch physician associations and patient associations, ensuring a   patient-centered approach | NR  During Visit | Complete an online survey 1 month after they visit the outpatient clinic and interact with the portal. |

| **I D** | **Author Year** | **Intervention**  Name/Picture | **Format** | **Description** | **Comparison** | **Stakeholders' Roles** | **Duration** | **Follow-up** |
| --- | --- | --- | --- | --- | --- | --- | --- | --- |
| 9 | Karikos ki et al. (2023)  [32] | Oral Health Promotion Intervention (OHPI) | Counseling, home delivery of toothpaste and toothbrushes, written information | The OHPI included counseling by motivational interviewing, home-delivered toothpaste and toothbrushes, and written information, and was provided at baseline, 6, 12, and 18 months of age to the CHD-I group.  The primary outcome measure at 24 months was the child’s oral health behavior (toothbrushing, sugar intake, and dental care contact). The secondary outcome measures were parents’ awareness of the importance of oral health behavior and oral health behavior as a predictor for child behavior. | Compared with the children were randomly assigned to usual primary oral health care only.  Also, a parallel passive control (C) group of 87 healthy children was recruited at birth. | **Children with CHD:**   - Recipients of the intervention. - They are indirectly involved through their parents/guardians who implement oral health promotion strategies.   **Parents/Guardians:**   - Responsible for implementing the intervention strategies at home. - Participated in counseling sessions, received guidance on maintaining good oral health in CHD children, and ensured their adherence to oral health practices. **Dental Hygienist (E.K.):** - Led the counseling sessions. - - Provided oral health education, motivational interviewing, and guidance on proper dental care practices tailored to CHD children. - Available for consultation throughout the intervention period.   **Specialist Dentist in Pedodontics:**   - Available for phone consultation, providing advice and support as needed. **Local Primary Care Health Clinic:** - Contacted to promote the formation of dental care contact for CHD children. - Being informed about the study and encouraged to support oral health initiatives for children under their care. | One and a half year intervention, with sessions at baseline, 6, 12,  and 18 months. | Follow-up assessment at 24 months. |
| 1  0 | Klause n et al. (2016)  [33] | The Paediatric Rehabilitation for Vanguard in Lifeskills (PReVaiL) | A 52-week eHealth program utilizing the Internet, mobile applications, and SMS-based platforms. | PReVaiL consisted of individually tailored eHealth messages encouraging physical activity for 52 weeks. All patients received 45 minutes of group-based health education and 15 minutes of individual counseling involving patients' parents. The experimental intervention was a 52-week Internet, mobile application, and  SMS-based program delivering individually tailored text messages to encourage physical activity. | Allocated to a control intervention (n=77)   - Received allocated intervention (n=77) - Did not receive allocated intervention (n=0) | **Researchers:** Designed the study, conducted data analysis, interpreted results, and disseminated findings. **Healthcare Providers** (Cardiologists, Nurses, Exercise Physiologists/ Physiotherapists, Psychologists/Social Workers, and Coach Specializing in Adolescence): - Delivered health education and individual counseling to participants, monitored participant progress, and provided support.  **Adolescents with CHD:** Engaged with the eHealth intervention, recorded physical activity data, and responded to motivational messages.  **Parents/Caregivers:** Provided support to adolescents participating in the intervention, possibly assisted in navigating the eHealth platform, and encouraged adherence to the intervention. | 52 Weeks | 1 Year |

| **I D** | **Author Year** | **Intervention**  Name/Picture | **Format** | **Description** | **Comparison** | **Stakeholders' Roles** | **Duration** | **Follow-up** |
| --- | --- | --- | --- | --- | --- | --- | --- | --- |
| 1  1 | Lemire et al. (2020)  [34] | Evidence-based  , multi-faceted physical activity intervention | Printed materials, physical activity toolkit, website | The intervention includes posters in clinic rooms, a physical activity toolkit used by clinicians, an introduction to a website about physical activity and CHD, and discussions about physical activity during clinic visits. Clinicians undergo training to deliver the intervention. Patients receive counseling on 5 key physical activity messages, have their questions addressed, and gain access to a personalized website for activity suggestions and support from a Registered Kinesiologist. | Usual care | **Clinicians:** Deliver physical activity counseling during clinic visits.  **Allied Health Professionals:** Provide assistance in intervention delivery.  **Registered Kinesiologist:** Offer support and guidance to participants.  **Parents:** Support/encourage children's participation in physical activity.  **Children:** Engage in discussions and activities promoting physical activity. | NR | 6 Months |

**Table S4.** Outcome specification.

| **I D** | **Author Year** | **Outcome Measures** | **Intervention Group V.S. Control Group** | **Statistical Analysis** | **Engageme nt** Utilization Satisfaction | **Children's Health Literacy** (Understanding of CHD)  (Self-management) (Coping) (Empowerment) (Healthcare Utilization) (Health Outcome) | **Parental Support** (Educational Support) (Emotional Support) (Caregiving Support) (Financial Support) | **Provider Efficiency** (Save Time/Effort) (Treatment Adherence) (Care Coordination) (Shared Decision-Making) (Patient/Family Satisfaction) |
| --- | --- | --- | --- | --- | --- | --- | --- | --- |
| 1 | Ni et al. (2016)  [36] | - Caregiving Knowledge   (Repeated Measures ANOVA, Post hoc t-tests)   - Caring Behaviors   (Repeated Measures ANOVA, Post hoc t-tests)   - Self-Efficacy   (Repeated Measures ANOVA, Post hoc t-tests)   - Left Ventricular Ejection Fraction (LVEF) (Repeated Measures ANOVA, Post hoc tests, Pearson's correlation analysis) - Peripheral Oxygen Saturation (SpO2) (Repeated Measures ANOVA, Post hoc tests, Pearson's correlation analysis) | Higher at 1 and 3 months  Higher at 1 and 3 months  Higher at 1 and 3 months Higher at 3 months Higher at 3 months | Software: SPSS - Demographic data: χ2 test & independent group t-test - Outcome analyzed used repeated measures analysis of variance (ANOVA) -  Post hoc t-tests used to assess intergroup differences at each time point (baseline, one month, and three months after surgery)  - Intragroup differences between time points were evaluated using one-way ANOVA where appropriate.- Significance: p < .05 | NR | (Health Outcome) | (Educational Support) (Emotional Support) (Caregiving Support).  Parents' knowledge, behaviors, and  self-efficacy in caring for their children with CHD correlate with better postoperative recovery outcomes. | (Care Coordination) (Shared  Decision-Making) |
|  |  | - New York Heart Association Disease Classification |  |  |  |  |  |  |
| 2 | Biglino et al. (2015)  [30] | Parent Assessment   - Self-assessed knowledge (before) - Self-assessed knowledge (after) - Clarity of explanation received | 7.9±1.6 8.1±1.7  9.1±1.1 9.0±1.2  9.3±1.1 9.1±1.3 | Questionnaires were administered to the participants before and after the visits and an additional questionnaire was administered to the attending cardiologist.  Differences between continuous variables in the 2 groups assessed with a two-sample Student t-test and differences between groups in terms of sex (male vs female) and education (low vs high, with high defined as university graduate + university postgraduate) were assessed with χ2 test. Analysis was performed in Stata (V 13.1, College Station, Texas, USA).  Software: Stata | Parents and cardiologist s found the models to be very useful and helpful in engaging parents in discussing congenital heart defects. | NR | (Educational Support)  Parental knowledge was not associated with their level of education (p=0.2) and did not improve following their visit. | (Save Time/Effort)  On average, consultations involving 3D models lasted  5 min longer (p=0.02). |
|  |  | - Usefulness of the 3D model | 9.5±0.7 – |  |  |  |  |  |
|  |  | Cardiologist Assessment  - Parent knowledge (after) | 7.0±1.9 8.0±1.7 |  |  |  |  |  |
|  |  | - Quality of interaction with the model | 9.1±1.4 – |  |  |  |  |  |
|  |  | - Usefulness of the 3D model | 8.8±1.1 – |  |  |  |  |  |
|  |  | Values scale 1–10, with 1 lowest score and 10 highest score.  Values are from the parents' and cardiologists' questionnaires.  Main outcome measures Rating (1–10) for the liking of the 3D model, its usefulness and the clarity of the explanation received were recorded, as well as rating (1–10) of the parental understanding and |  |  |  |  |  |  |

| **I D** | **Author Year** | **Outcome Measures** | **Intervention Group V.S. Control Group** | **Statistical Analysis** | **Engageme nt** Utilization Satisfaction | **Children's Health Literacy** (Understanding of CHD)  (Self-management) (Coping) (Empowerment) (Healthcare Utilization) (Health Outcome) | **Parental Support** (Educational Support) (Emotional Support) (Caregiving Support) (Financial Support) | **Provider Efficiency** (Save Time/Effort) (Treatment Adherence) (Care Coordination) (Shared Decision-Making) (Patient/Family Satisfaction) |
| --- | --- | --- | --- | --- | --- | --- | --- | --- |
|  |  | their engagement according to the cardiologist. Furthermore, parental knowledge was assessed by asking them to mark diagrams, tick keywords and provide free text answers. The duration of consultations was recorded and parent feedback collected. |  |  |  |  |  |  |
| 3 | Amedr o et al. (2019)  [29] | Primary Outcome Measure:   - Quality of life score: PedsQL   self-questionnaire (version 13–18 yrs for adolescents and version 18–25 yrs for young adults)  Secondary Outcome Measures:   - CPET variables: VO2max, VAT, VE/VCO2 slope, OUES, and oxygen pulse - Peak exercise stroke volume (PhysioFlow®) - Level of physical activity (Ricci and Gagnon questionnaire) - Level of knowledge (Leuven knowledge CHD questionnaire) - Clinical outcomes: NYHA functional class, blood pressure, body mass index (BMI), healthcare usage (primary and secondary care contacts, hospitalization), and medication - Level of anxiety (STAI self-questionnaire for young adults and the STAI-Children   self-questionnaire for adolescents)   - Level of depression (BDI self-questionnaire for young adults and CDI self-questionnaire for adolescents) - Proxy version of the PedsQL for parents of adolescents (aged 13–18 years old) - The socio-economic status of the patient and/or the family (only at baseline) - Safety outcomes - Acceptability of the intervention to participants | NR | NR  Intention-to-treat (ITT) analysis. | NR | NR | NR | NR |

| **I D** | **Author Year** | **Outcome Measures** | **Intervention Group V.S. Control Group** | **Statistical Analysis** | **Engageme nt** Utilization Satisfaction | **Children's Health Literacy** (Understanding of CHD)  (Self-management) (Coping) (Empowerment) (Healthcare Utilization) (Health Outcome) | **Parental Support** (Educational Support) (Emotional Support) (Caregiving Support) (Financial Support) | **Provider Efficiency** (Save Time/Effort) (Treatment Adherence) (Care Coordination) (Shared Decision-Making) (Patient/Family Satisfaction) |
| --- | --- | --- | --- | --- | --- | --- | --- | --- |
| 4 | van der Mheen et al. (2018)  [38] | Primary Outcome Measures:   - Child Behavioral/Emotional Problems (CBCL) - Symptom Checklist-90-Revised (SCL-90-R) - Parental Mental Health (SCL-90-R) Secondary Outcome Measures: - School Days Sick/Absent (Rotterdam Quality of Life Interview) - Disease-Specific Knowledge and Illness Perception (Rotterdam Knowledge Questionnaire) - School Functioning (Teacher Report Form - TRF) - Executive Functioning (Behavior Rating Inventory of Executive Functioning - BRIEF) - Enjoyment of Physical Activity (Groningen Enjoyment Questionnaire) - Parental Worry (Penn State Worry Questionnaire - PSWQ) - Parenting Stress (Nijmeegse Ouderlijke Stress Index verkort - NOSIK) - Quality of Life of Child and Sibling (Child Health Questionnaire Parent Form-50 - CHQ-PF50) - Parental Quality of Life (Short-form 36 Health Survey - SF-36) - Family Functioning (Family Assessment Device, general functioning subscale - FAD) - Social Validity (Questionnaire on Satisfaction, Attendance, and Completion of CHIP-Family) - Demographic Variables (Age, Gender, Socio-economic Status) - Medical Variables (Cardiac Diagnosis, Surgery, Intrusive Procedures) | NR | To test the effectiveness of CHIP-Family on the primary outcome measures (for parents: mental health [SCL-90-R]; for children: behavioral/emotional problems [CBCL]), repeated  measures ANOVAs will be used for parental and child outcomes separately. Group (CHIP-Family versus CAU) will be the  between-subjects variable, and assessment (T1 versus T2) will be the within-subjects variable. Likewise, repeated measures ANOVAs will be conducted for the secondary outcome measures. Additional regression analyses will be conducted to investigate in what way demographic factors, medical factors, and life events moderate the effect of  CHIP-Family on the primary outcome measures.  T1: Baseline assessment  T2: Follow-up assessment (6 months after T1) | NR | NR | NR | NR |

| **I D** | **Author Year** | **Outcome Measures** | **Intervention Group V.S. Control Group** | **Statistical Analysis** | **Engageme nt** Utilization Satisfaction | **Children's Health Literacy** (Understanding of CHD)  (Self-management) (Coping) (Empowerment) (Healthcare Utilization) (Health Outcome) | **Parental Support** (Educational Support) (Emotional Support) (Caregiving Support) (Financial Support) | **Provider Efficiency** (Save Time/Effort) (Treatment Adherence) (Care Coordination) (Shared Decision-Making) (Patient/Family Satisfaction) |
| --- | --- | --- | --- | --- | --- | --- | --- | --- |
|  |  | Life Events (Life Events Subscale of the Cognitive Emotion Regulation Questionnaire - Child Version)  Predictors: Demographic variables Medical variables  Life events  Sample size calculation |  |  |  |  |  |  |
| 5 | Uzark et al. (1982)  [37] | Mood Indice (Johnson-Radloff and Helmreich Mood Adjective Checklist (MACL)  Knowledge Scores (ten questions related to hospitalization events and sensations)  Catheterization Behavior (Child's behavior during catheterization rated by two technicians)  Post-discharge Questionnaires (Parents completed a questionnaire assessing child's adjustment post-discharge) | The inverse relationship was observed between parental upset (anger index) and the child's happiness (cheerfulness index; P = .0008). No significant relationship was found between viewing the videotape and parent/child mood.  Children in the experimental had significantly greater knowledge (M = 76.19) compared to the control (M = 58.00; P < .0001).  Children in the experimental slept more during the procedure (M = 3.05) compared to the control (M = 2.30; P = .05). Children in the experimental asked more questions (M = 2.27) than the control (M = 1.55; P = .02).  No significant differences between groups in sleep problems post-hospitalization. Trend (P = .06) towards more positive adjustment in the experimental group, with increased independence reported. | - Analysis of covariance (ANCOVA) used to compare grouped data for knowledge scores and catheterization laboratory behavior.- Mood indices were constructed using  single-linkage cluster analysis and interitem reliability of the item clusters. - Correlation analysis (r) was used to examine the relationship between parental upset (anger index) and the child's happiness (cheerfulness index). - T-tests were used to compare differences in parental fear between the experimental and control groups. - Chi-square was used to assess differences in post-hospitalization behavioral changes between the two groups. - Stepwise regression analysis was employed to select covariates for each test of treatment effects on outcome measures. -  One-way analysis of covariance used to analyze the impact of the videotapes, controlling for covariates such as age and mood variables. | NR | (Understanding of CHD)  (Self-management) (Coping) (Empowerment) (Health Outcome)  These statistical methods were used to evaluate the effectiveness of the intervention (videotape viewing) on children's knowledge, mood, and behavior during hospitalization for cardiac catheterization. | (Educational Support) (Emotional Support) (Caregiving Support) | NR |

| **I D** | **Author Year** | **Outcome Measures** | **Intervention Group V.S. Control Group** | **Statistical Analysis** | **Engageme nt** Utilization Satisfaction | **Children's Health Literacy** (Understanding of CHD)  (Self-management) (Coping) (Empowerment) (Healthcare Utilization) (Health Outcome) | **Parental Support** (Educational Support) (Emotional Support) (Caregiving Support) (Financial Support) | **Provider Efficiency** (Save Time/Effort) (Treatment Adherence) (Care Coordination) (Shared Decision-Making) (Patient/Family Satisfaction) |
| --- | --- | --- | --- | --- | --- | --- | --- | --- |
| 6 | Zablah et al. (2021)  [39] | Pre:  Understanding of Specific Cardiac Anatomy: Understanding of the Treatment Plan:  Overall Understanding of Patient's CHD:  Previous knowledge about 3DPM among parents  Familiarity with 3DPM in explanation of heart condition  Interest in experiencing the 3D printed model of child's heart  Claimed understanding of 3DPM representations before physician's explanation  Post:  Understanding of 3DPM representations after detailed discussion with physician  Satisfaction of the explanation of 3DPM representations  Disease understanding after discussion with physician  Agreement on ease of understanding using 3DPM  Preferred data source for complex discussions with physicians  Overal:  Improvement in understanding and satisfaction post-intervention | Likert: between 3 (34.8%) and  4 (32.6%)  Likert: 3 (34.8%) and 4  (39.2%)  Likert: 3 (28.3%) and 4  (28.3%)  43.2%  11.4%  Likert: between 4 (28.3%) and  5 (60.9%)  71.7%  Likert: between 4 (34.8%) and  5 (56.5%)  Likert scale: 5 (73.9%)  Likert scale: 4 (37%) - 5  (58.7%)  Strong agreement (87%)  Preferred: 3DPM (84.8%)  Significant improvement (p < 0.0001) | Software: SPSS. - Categorical data summarized by frequency and percentage. Continuous data were shown as median and range. - Wilcoxon signed-rank test and/or Mann-Whitney U were used to compare responses about the level of understanding between traditional imaging and 3DPM. - A p-value of  0.05 was established as significant. NR  Pre and Post Assessments | Satisfaction | (Understanding of CHD) | (Educational Support) | (Care Coordination) (Patient/Family Satisfaction) |
| 7 | Liddle et al. (2022)  [35] | Improvement in Medical Knowledge | Pre-intervention: 47.7% had a class I understanding of their heart defect; post-intervention: increased to 88.6% | A weighted Cohen’s kappa statistic was used to determine inter-rater reliability for the medical knowledge scoring. Inter-rater reliability was | Satisfaction | (Understanding of CHD) | (Educational Support) | (Care Coordination) (Patient/Family Satisfaction) |

| **I D** | **Author Year** | **Outcome Measures** | **Intervention Group V.S. Control Group** | **Statistical Analysis** | **Engageme nt** Utilization Satisfaction | **Children's Health Literacy** (Understanding of CHD)  (Self-management) (Coping) (Empowerment) (Healthcare Utilization) (Health Outcome) | **Parental Support** (Educational Support) (Emotional Support) (Caregiving Support) (Financial Support) | **Provider Efficiency** (Save Time/Effort) (Treatment Adherence) (Care Coordination) (Shared Decision-Making) (Patient/Family Satisfaction) |
| --- | --- | --- | --- | --- | --- | --- | --- | --- |
|  |  | Inter-rater Reliability  Qualitative Feedback | Pre-intervention: 18.5% had a class I understanding of their surgical history;  post-intervention: increased to 68.2%  Substantial agreement for cardiac defect (kappa score: 0.75) and surgery knowledge (kappa score: 0.74) classification  Patients/family appreciated the ability to visualize the patient's CHD | interpreted as follows: 0.01–0.20 as none to slight, 0.21–0.40 as fair, 0.41–0.60 as moderate, 0.61–0.80 as substantial, and 0.81–1.00 as almost perfect agreement. A Wilcoxon signed rank test was calculated to determine the statistical difference in medical knowledge before and after the educational intervention.  p-Values < 0.05 were considered statistically significant. |  |  |  |  |
| 8 | Etnel et al. (2017)  [31] | Disease-specific knowledge Anxiety and depression Mental quality of life  Patient/caregiver involvement and autonomy  Experiences with and views on patient information  Views on participation in decision-making Decisional conflict  surveys and interviews among patients:first phase | NR | NR | NR | NR | NR | NR |
| 9 | Karikos ki et al. (2023)  [32] | Primary Outcome:  Child’s Oral Health Behavior (This includes toothbrushing frequency, sugar intake, and dental care contact) | - At 24 months, toothbrushing was performed twice a day in 20/27 (74%) among CHD-I, in 13/30 (43%) among CHD-C (CHD-I vs. CHD-C p = 0.03),   and in 37/50 (74%) among healthy comparisons (CHD-C vs. C p = 0.01).   - Electric toothbrush use was 12/27 (44%) in CHD-I, 5/30 | t-test: Used to compare mean values between groups. - Mann-Whitney U test: Used to compare median values between groups. - Fisher’s exact test: Used to compare child and parent oral health behavior outcome variables, as well as sociodemographic characteristics between groups. - Simple logistic regression: Used to estimate | NR | (Self-management) (Health Outcome) | (Educational Support) (Caregiving Support) | NR |

| **I D** | **Author Year** | **Outcome Measures** | **Intervention Group V.S. Control Group** | **Statistical Analysis** | **Engageme nt** Utilization Satisfaction | **Children's Health Literacy** (Understanding of CHD)  (Self-management) (Coping) (Empowerment) (Healthcare Utilization) (Health Outcome) | **Parental Support** (Educational Support) (Emotional Support) (Caregiving Support) (Financial Support) | **Provider Efficiency** (Save Time/Effort) (Treatment Adherence) (Care Coordination) (Shared Decision-Making) (Patient/Family Satisfaction) |
| --- | --- | --- | --- | --- | --- | --- | --- | --- |
|  |  | Secondary Outcome Measures:  Parents' Awareness of the Importance of Oral Health Behavior: (Assesses the level of understanding and knowledge among parents regarding the significance of oral health practices for children with CHD.)  Oral Health Behavior as a Predictor for Child Behavior: (Investigates whether parental oral health behavior influences the oral health behavior of their children.) | (17%) in CHD-C (CHD-I vs. CHD-C p = 0.04), and 7/50  (14%) in healthy comparison (CHD-C vs. C p = 0.76)  children.   - Toothbrushing and use of electric toothbrushes improved between 12 months and 24 months in the CHD-I group. - Sugary drink intake was more common among CHD-C (CHD-C vs. C p = 0.02), but   comparable to CHD-I children.   - There were no statistical group differences in dental care contact.   Parental toothbrushing positively correlated with children's toothbrushing frequency, highlighting the role of parental oral hygiene habits in shaping children's oral health behavior.  Parental toothbrushing predicted child toothbrushing twice a day. | associations between the CHD and comparison groups, presented as odds ratios with 95% confidence intervals. - Multiple (adjusted) logistic regression: Used to investigate the effect of CHD-related background variables and CHD study group on the main outcome variables among CHD children, as well as the effect of parental oral health behavior and study group on the main outcome variables among children in the CHD and comparison groups.  motivational interviewing technique |  |  |  |  |
| 10 | Klause n et al. (2016)  [33] | Primary Outcomes  Peak oxygen uptake (VO2 peak) - Assessed via an incremental cardiorespiratory exercise test on a bicycle ergometer.) | (Mean VO2 peak at 1 year): (Mean ± SD)  Intervention Group: 43.2 ± 9.7 ml·kg−1·min−1  Control Group: 46.2 ± 10.1 ml·kg−1·min−1  Between-Group Difference:  -0.65 (95% CI -2.66 to 1.36),  P Value: 0.52  *Subgroup analyses by gender, per-protocol recipients, and baseline VO2 peak did not reveal statistically significant differences. | Primary Analysis for Continuous Outcomes: Covariance (ANCOVA) Adjusted for: VO2 peak at baseline. Stratification variables (gender and high/low exercise capacity) - Logistic Regression adjusted for stratification variables. -Primary Analysis for Binary Outcomes: Logistic Regression  Adjusted for stratification variables. - Secondary Analysis for Continuous Outcomes:  ANCOVA adjusted for:  VO2 peak at baseline. Stratification variables (gender and high/low exercise capacity). Age at | NR | (Self-management) (Health Outcome) | NR | NR |

| **I D** | **Author Year** | **Outcome Measures** | **Intervention Group V.S. Control Group** | **Statistical Analysis** | **Engageme nt** Utilization Satisfaction | **Children's Health Literacy** (Understanding of CHD)  (Self-management) (Coping) (Empowerment) (Healthcare Utilization) (Health Outcome) | **Parental Support** (Educational Support) (Emotional Support) (Caregiving Support) (Financial Support) | **Provider Efficiency** (Save Time/Effort) (Treatment Adherence) (Care Coordination) (Shared Decision-Making) (Patient/Family Satisfaction) |
| --- | --- | --- | --- | --- | --- | --- | --- | --- |
|  |  | Secondary Outcomes  Physical Activity (MVPA) (Mean daily minutes of moderate to vigorous physical activity.)  *Physical activity assessed by accelerometer.  *Physical activity assessed by electronic questionnaire.  Health-related Quality of Life  Assessed by the Paediatric Quality of Life Inventory.   - Generic module: Physical, emotional, social, school functioning. - Disease-specific module: Heart problems, treatment, physical appearance, treatment anxiety, cognitive problems, communication.   *Primary Outcome (VO2 peak): p = 0.52 Secondary Outcome (Physical Activity): p > 0.05 (not significant)  Secondary Outcome (Health-related Quality of Life   - Generic): p > 0.05 (not significant)   Secondary Outcome (Health-related Quality of Life   - Disease-specific): p > 0.05 (not significant) | Intervention Group: 40.3  minutes (SD 21.8)  Control Group: 41.3 minutes (SD 22.9)  *Between-group difference:  -0.04 min/day (95% CI -2.23 to 0.23), not significant.  Generic HRQoL:  Intervention Group: 80.0 ± 9.4  Control Group: 80.4 ± 9.5  Disease-specific HRQoL: Intervention Group: 85.2 ± 10.7  Control Group: 84.6 ± 9.7  *Differences in generic and disease-specific health-related quality of life between intervention and control groups were insignificant. | test years. Lung function. Muscle strength  Body composition.- Secondary Analysis for Binary Outcomes: Unadjusted Logistic Regression. - Tertiary Analysis for Continuous Outcomes: ANCOVA  Adjusted for: Baseline values, Stratification variables, Cluster association. - Tertiary Analysis for Binary Outcomes: Logistic Regression Adjusted for stratification variables and cluster association.  mentioned "The investigator read the items to patients and recorded their responses…" So, it could mean that the data collected by interviews. |  |  |  |  |
| 11 | Lemire et al. (2020)  [34] | Primary Outcome:  Daily physical activity (number of steps, minutes of moderate-to-vigorous activity) assessed via pedometer one week per month for 6 months.  Pedometer (PiezoRX)  Secondary Outcome:  Physical Activity Motivation (CSAPPA)  Physical Literacy. Quality of life (PedsQL). | Repeated Measures ANOVA: Compares average daily steps and moderate-to-vigorous activity between study groups over time. Adjusts for potential confounders such as age, sex, diagnosis, ventriculotomy, and age at repair.  ANCOVA compares study group means, adjusting for baseline measures.  Logistic regression compares study group odds ratios.  ANCOVA compares study group means, adjusting for baseline measures. | Primary Outcome (Physical Activity Participation):  Repeated Measures ANOVA: Compares average daily steps and moderate-to-vigorous activity between study groups over time. ANCOVA: Adjusts for potential confounders such as age, sex, diagnosis, ventriculotomy, and age at repair.  Secondary Outcomes:  Quality of Life (PedsQL): ANCOVA compares study group means, adjusting for baseline measures.  Physical Literacy Screening: Logistic regression compares study group odds ratios.  Physical Activity Motivation (CSAPPA): ANCOVA compares study group means, adjusting for | NR | NR | NR | NR |

| **I D** | **Author Year** | **Outcome Measures** | **Intervention Group V.S. Control Group** | **Statistical Analysis** | **Engageme nt** Utilization Satisfaction | **Children's Health Literacy** (Understanding of CHD)  (Self-management) (Coping) (Empowerment) (Healthcare Utilization) (Health Outcome) | **Parental Support** (Educational Support) (Emotional Support) (Caregiving Support) (Financial Support) | **Provider Efficiency** (Save Time/Effort) (Treatment Adherence) (Care Coordination) (Shared Decision-Making) (Patient/Family Satisfaction) |
| --- | --- | --- | --- | --- | --- | --- | --- | --- |
|  |  | Health System Impacts:  Clinic Visit Time:  Clinic Contacts:  Intervention Delivery and Additional Support: | Mann-Whitney U-Test compares medians between study groups.  Poisson's model compares study groups with respect to the rate of physical activity encounters.  Proportions and 95% confidence intervals evaluate the probability of the intervention group receiving counseling and requiring additional support. | baseline measures. Health System Impacts:  Clinic Visit Time: Mann-Whitney U-Test compares medians between study groups.  Clinic Contacts: Poisson model compares study groups with respect to the rate of physical activity encounters.  Intervention Delivery and Additional Support: Proportions and 95% confidence intervals evaluate the probability of intervention group receiving counseling and requiring additional support. |  |  |  |  |

**Table S5.** Summary of studies.

| **Author**  **- Ref** | **Date** | **Country** | **Study Design** | **Study Design Detail** | **Pediatric CHD**  **Children Age** | **Parent s Age Range** | **Intervention Tested on** | **Sample Size** | **Intervention Duration** | **Follow-U p Time** | **Setting** | **Intervention** | **Purpose** |
| --- | --- | --- | --- | --- | --- | --- | --- | --- | --- | --- | --- | --- | --- |
| Uzark et al. [37] | 1982 | USA | (RCT) | Prospective Randomized Clinical Trial | 3 to 12 years | N/A | Patients and Parents | (n=53)  Intervention (n=31) Control (n=22) | 16 Minutes | 4 To 6  Weeks after Hospital Discharge | Children's Hospital | Leonard Z. Lion Presents... Learning About Your Heart Catheterization  - A 16-minute Videotape Presentation about Cardiac Catheterization Hospitalization | The purpose of this prospective study was to evaluate the effect of the videotape on (1) the child's knowledge of the events and sensations experienced during hospitalization for cardiac catheterization and (2) the child's coping as evidenced by behavior during cardiac catheterization  and adjustment after hospitalization. |
| Biglino et al. [30] | 2015 | England | (RCT) | Questionnaire- Based. (randomization  ) - Feasibility and acceptability study | 6 to18years | 35 to 51year s | Parents | (n=103)  Intervention (n=45) Control (n=52) | Routine Outpatient Visits | NR | Cardiology Outpatient Clinic | 3D Patient-Specific Models of Congenital Heart Defects  - 3D Medical Models as Visual Aids during Cardiology Consultations | To assess the communication potential of 3D patient-specific models of congenital heart defects and their acceptability in clinical practice for cardiology consultations. |
| Ni et al.  [36] | 2016 | China | (RCT) | Prospective  Clinical Trial | 1 month to  5 years | N/A | Parents | (n=86)  Intervention (n=44) Control (n=42) | 40 Minutes and  3 Monthly 10 Minutes | 1 Month  and 3 Months after Surgery | Children’s  Hospital and Home | Empowerment-Based Health Education Program   - Face-to-Face Education Sessions - Follow-Up Telephone Calls | To evaluate the effectiveness of an empowerment health education  program for improving caregiving knowledge, caring behaviors, and self-efficacy of parents caring for children after corrective surgery for CHD. |
| Klausen et al. [33] | 2016 | Denmark | (RCT) | Randomized Clinical Trial | 13 to 16 years | N/A | Patients | (n=158)  Intervention: (n=77) Control: (n= 77) | 52 Weeks | 1 Year | Institute of Sports Medicine and Home | The Paediatric Rehabilitation for Vanguard in Lifeskills (PReVaiL)  - 52-week eHealth program, mobile application, and SMS-based platforms | Evaluate the effectiveness of a 52-week eHealth intervention, comprising the Internet, mobile application, and SMS-based components, on physical fitness, physical activity, and health-related quality of life in adolescents  with congenital heart disease (CHD). |
| Etnel et  al. [31] | 2017 | Netherland | (Pilot Study) +  (RCT) | Multicenter  Stepped-Wedge Implementation Trial | Teenagers  and Adult | N/A | Adolescents,  Adults, or Parents/Caregivers | Phase 1: (n=105)  Patients (n = 63) Caregiver (n = 10) Physicians (n = 32) Phase 2: (n=10) Patients (n = 2) Caregiver (n = 2) Physician (n = 6) Phase 3: (n=250)  Intervention (n = 125)  Control (n = 125) | No Limit | 1 Month | Congenital  Cardiac Centers and Home | Web-based Portal   - Online, Evidence-based Portal supporting Patients and Physicians. - Including information on diagnosis, treatment, prognosis, psychosocial aspects, and implications for daily life | The objective of this pilot project was to develop an online information  portal that aims to (1) improve patient knowledge and involvement and to subsequently reduce anxiety, depression, and decisional conflict and improve mental quality of life and (2) to support physicians in informing and communicating with their patients. |
| van der Mheen et al. [38] | 2018 | Netherland | (RCT) | Single-Center, Single-Blinded, Randomized Controlled Trial | 4 to 7 years | N/A | Patients and Parents | (n=90)  Intervention (n=45) Control (n=45) | One-day Workshop | 6 Months | Children’s Hospital | CHIP-Family Intervention   - Workshop (Together Parent Module and Child Module) - Workshop (Separate Parent Module and Child Module) | The CHIP-Family study aims to (1) test the effects of CHIP-Family on parental mental health and psychosocial wellbeing of CHD-children and to (2) identify baseline psychosocial and medical predictors for the effectiveness of CHIP-Family. |
| Amedro et al. [29] | 2019 | France | (RCT) | Prospective, Multicentre, Randomised, Controlled, Parallel Arm  Study | 13 to 25 years | N/A | Patients | (n=130)  Intervention (n=65) Control (n=65) | 12 Weeks | 12 Months | CHD Centers and Cardiac Rehabilitation Centers | The QUALI-REHAB Cardiac Rehabilitation Program   - Combined Center-based and Home-based training - Including personalized education, clinical examination, cardiopulmonary exercise testing, supervised interval training exercises | The study aimed to assess the impact of a combined center- and home-based cardiac rehabilitation program on the quality of life of adolescents and young adults with congenital heart disease. |
| Lemire et al. [34] | 2020 | Canada | (RCT) | Cluster Randomized Controlled Trial | 5 to 17 years | N/A | Patients and Parents | (n=200)  Intervention (n=100) Control (n=100) | 6 Months (3-days  workshop and conference call each month) | 6 Months | Pediatric Cardiology Clinics | Evidence-Based, Multi-Faceted Physical Activity Intervention  - Printed materials, physical activity toolkit, website | The goal is to determine whether providing resources and protocols enables clinicians to counsel about physical activity as part of every pediatric cardiology appointment. Evaluations of healthcare system impact and intervention delivery in small, medium, and large clinics will assess the applicability of this approach to all pediatric cardiac clinics. |
| Zablah et al. [39] | 2021 | USA | (Observational Study) | Single-Center Cross-Sectional Study | 1 month to  21 years | N/A | Parents | (n=46) | Routine Outpatient Visits | NR | Children's Hospital | Three-Dimensional Printed Models (3DPM)   - Patients and families educated about the child's cardiac anatomy and treatment plan - Empowering patients and families with essential knowledge before the procedure | This study reports on the experience using three-dimensional printed models (3DPM) for patient education and compares it with other methods for patients undergoing cardiac catheterizations. The goal is to improve  families' and patients' understanding of cardiac anatomy, increase patient satisfaction, and optimize doctor-patient communication. |
| Liddle et  al. [35] | 2022 | USA | (Observational  Study) | Prospective  pre-post study | 13 to 18  years | N/A | Patients | (n=22) | 30-Min  Tele-Education Session | NR | Pediatric Care  Center | Digital 3D Heart Models   - Tele-education sessions with pediatric cardiologist - Display of patient-specific 3D heart models using Cardiac Review 3D software - Patients received a USB drive with 3D heart video and digital files for printing | Investigate the feasibility of using digital 3D models with tele-education  for adolescents with CHD |
| Karikos ki et al. [32] | 2023 | Finland | (RCT) | Randomized Controlled Trial (National Population-Bas  ed) | 0 to 24 months | N/A | Patients and Parents | (n=72)  Intervention: (n=35) Control: (n=37) | 1.5 Years  (sessions at 6,  12, 18 months | 24 Months | Children’s Hospital and Home | Oral Health Promotion Intervention (OHPI)  - Counseling, home delivery of toothpaste and toothbrushes, written information | To investigate the effectiveness of repeat counseling provided by a dental hygienist in improving oral health behavior in children with major CHD during the first 1,000 days of life. |
