# Supplementary material for: Exploring Health Educational Interventions for Children With Congenital Heart Disease: Scoping Review
Source: JMIR Pediatr Parent. 2025 Jan 24;8:e64814. doi: 10.2196/64814 (PMC11806270; doi:10.2196/64814)
Supplement: Multimedia Appendix 4 [file pediatrics_v8i1e64814_app4.docx]

1. Winder MM, Marietta J, Kerr LM, Puchalski MD, Zhang C, Ware AL, Cowley CG. Reducing Unnecessary Diagnostic Testing in Pediatric Syncope: A Quality Improvement Initiative. Springer 2021;42(4):942–950. doi: 10.1007/s00246-021-02567-4

2. Toledo JR, Hughes H, Sims J. Management of non-compliance to medical regimen: A suggested methodological approach. INT J HEALTH EDUC 1979;22(4):232–241. Available from: https://www.scopus.com/inward/record.uri?eid=2-s2.0-0018621728&partnerID=40&md5=58fe8f2b8c4cb1c77122c3a1744bfd03

3. Taitson PF, Kwong DD, Lima GCA, Coelho LS, Bruce WD, Bernardes NO. Incidence and anatomy of cardiac malformations in children conceived by assisted reproduction techniques - A review. SBRA - Associação Brasileira de Reprodução Assistida (Brazilian Society of Assisted Reproduction) 2014;18(2):52–54. doi: 10.5935/1518-0557.20140005

4. Purcell, , C. Preparation of school-age children and their parents for intensive care following cardiac surgery. Churchill Livingstone 1996;12(4):218–225. doi: 10.1016/S0964-3397(96)80085-1

5. Pollard MA, Curzon ME. The effectiveness of the yellow card warning system for paediatric cardiology patients. Community dental health England; 1992;9(4):391–2. Available from: http://ovidsp.ovid.com/ovidweb.cgi?T=JS&PAGE=reference&D=med3&NEWS=N&AN=1486527

6. Poh Soon JosephNgGong X, NarinderjitSam TH, HuaPhan KY. Beyond Your Sight Using Metaverse Immersive Vision With Technology Behaviour Model. J Cases Inf Technol 2023 Apr;25(1):1. doi: 10.4018/JCIT.321657

7. Peterson, M C. Preparation of the cardiac child and the family for surgery. Issues in comprehensive pediatric nursing England; 1979;3(7):61–71. doi: 10.3109/01460867909098876

8. Pelletier, , L. Collecting information: a way to cope with cardiac surgery. Maternal-child nursing journal United States; 1981;10(2):143–54. Available from: http://ovidsp.ovid.com/ovidweb.cgi?T=JS&PAGE=reference&D=med2&NEWS=N&AN=6909478

9. Nikolaidou Z, Bellander T. Health literacy as knowledge construction: Learning about health by expanding objects and crossing boundaries in networked activities. Elsevier Ltd 2020;24. doi: 10.1016/j.lcsi.2018.11.003

10. Nabuco O. Delivering Deep Health Information Using Clinical Eye. Int J Web Portals 2013 Jan;5(1):28. doi: 10.4018/jwp.2013010103

11. Moreland P, Santacroce SJ. Illness Uncertainty and Posttraumatic Stress in Young Adults with Congenital Heart Disease. Lippincott Williams and Wilkins 2018;33(4):356–362. doi: 10.1097/JCN.0000000000000471

12. Monagle P, Newall F. Anticoagulation in children. Thromb Res 2012;130(2):142–146. doi: 10.1016/j.thromres.2012.03.028

13. Lowery CL, Bronstein JM, Benton TL, Fletcher DA. Distributing medical expertise: The evolution and impact of telemedicine in Arkansas. Health Aff 2014;33(2):235–243. doi: 10.1377/hlthaff.2013.1001

14. Jain VK, Nalini P, Chandra R, Srinivasan S. Congenital Malformations, Reproductive Wastage and Consanguineous Mating. Aust New Zealand J Obstet Gynaecol 1993;33(1):33–36. doi: 10.1111/j.1479-828X.1993.tb02048.x

15. Holland JE, DeMaso DR, Rosoklija I, Johnson KL, Manning D, Bellows AL, Bauer SB. Self-cathing experience journal: Enhancing the patient and family experience in clean intermittent catheterization. Journal of pediatric urology England; 2015;11(4):187. doi: 10.1016/j.jpurol.2015.03.011

16. Harden BW, Martin GR, Bradshaw EA. False-negative pulse oximetry screening for critical congenital heart disease: The case for parent education. Pediatr Cardiol 2013;34(7):1736–1738. doi: 10.1007/s00246-012-0414-5

17. Guo C, HuiZhou L, YouQiongChen H. Top-Tier Maternity and Children Specialist Smart Medical Care and Innovative 5G Outpatient Service Model. Complex 2021 Jan;2021. doi: 10.1155/2021/7270578

18. Farr SL, Downing KF, Riehle-Colarusso T, Abarbanell G. Functional limitations and educational needs among children and adolescents with heart disease. Blackwell Publishing Ltd 2018;13(4):633–639. doi: 10.1111/chd.12621

19. Downing KF, Espinoza L, Oster ME, Farr SL. Preventive Dental Care and Oral Health of Children and Adolescents With and Without Heart Conditions - United States, 2016-2019. MMWR: Morbidity & Mortality Weekly Report Atlanta, Georgia; 2022 Feb 11;71(6):189–195. doi: 10.15585/mmwr.mm7106a1

20. Diaz LK, Jones L. Sedating the Child with Congenital Heart Disease. Anesthesiol Clin 2009;27(2):301–319. doi: 10.1016/j.anclin.2009.05.003

21. Chatterjee A, Das D, Kohli P, Das R, Kohli V. Awareness of infective endocarditis prophylaxis and dental hygiene in cardiac patients after physician contact. Indian journal of pediatrics India; 2004;71(2):184. doi: 10.1007/BF02723109

22. Bellander T, Landqvist M. Becoming the expert constructing health knowledge in epistemic communities online. Routledge 2020;23(4):507–522. doi: 10.1080/1369118X.2018.1518474

23. Bajolle F, Lasne D, Elie C, Cheurfi R, Grazioli A, Traore M, Souillard P, Boudjemline Y, Jourdain P, Bonnet D. Home point-of-care international normalised ratio monitoring sustained by a non-selective educational program in children. Thromb Haemost 2012;108(4):710–718. doi: 10.1160/TH12-05-306

24. Zhao J, Chen S, Guo J, Wang Y. Sequential psychological care and health education for the rehabilitation of pediatric patients with congenital heart disease undergoing interventional occlusion. Asian journal of surgery Netherlands; 2024;47(2):1252–1253. doi: 10.1016/j.asjsur.2023.11.054

25. Zhang Q-L, Lin S-H, Lin W-H, Chen Q, Cao H. The effect of applying telehealth education to home care of infants after congenital heart disease surgery. International journal for quality in health care : journal of the International Society for Quality in Health Care England; 2023;35(1). doi: 10.1093/intqhc/mzac102

26. Yu X-R, Xie W-P, Liu J-F, Dai W-S, Cao H, Chen Q. Effect of WeChat follow-up education on breastfeeding efficiency of infants after congenital cardiac surgery and parents’ satisfaction: A short-term follow-up study. John Wiley and Sons Inc 2021;57(1):1899–1904. doi: 10.1111/jpc.15605

27. Yang H-L, Chen Y-C, Wang J-K, Gau B-S, Chen C-W, Moons P. Measuring knowledge of patients with congenital heart disease and their parents: Validity of the “Leuven Knowledge Questionnaire for Congenital Heart Disease.” Eur J Cardiovasc Nurs 2012;11(1):77–84. doi: 10.1177/1474515111429662

28. White SC, Sedler J, Jones TW, Seckeler M. Utility of three-dimensional models in resident education on simple and complex intracardiac congenital heart defects. Blackwell Publishing Ltd 2018;13(6):1045–1049. doi: 10.1111/chd.12673

29. Wernovsky G, Rome JJ, Tabbutt S, Rychik J, Cohen MS, Paridon SM, Webb G, Dodds KM, Gallagher MA, Fleck DA, Spray TL, Vetter VL, Gleason MM. Guidelines for the outpatient management of complex congenital heart disease. Congenit Heart Dis 2006;1(1):10–26. doi: 10.1111/j.1747-0803.2006.00002.x

30. Wernovsky G, Lihn SL, Olen MM. Creating a lesion-specific roadmap for ambulatory care following surgery for complex congenital cardiac disease. Cambridge University Press 2017;27(4):648–662. doi: 10.1017/S1047951116000974

31. Werner O, Bredy C, Lavastre K, Guillaumont S, De La Villeon G, Vincenti M, Gerl C, Dulac Y, Souletie N, Acar P, Pages L, Picot M-C, Bourrel G, Oude Engberink A, Million E, Abassi H, Amedro P. Impact of a transition education program on health-related quality of life in pediatric patients with congenital heart disease: study design for a randomised controlled trial. Health and quality of life outcomes England; 2021;19(1):23. doi: 10.1186/s12955-021-01668-1

32. Uzark K, VonBargen-Mazza P, Messiter E. Health education needs of adolescents with Congenital Heart Disease. J Pediatr Health Care 1989;3(3):137–143. doi: 10.1016/0891-5245(89)90063-1

33. Uzark K, Collins J, Meisenhelder K, Dick M, Rosenthal A. Primary preventive health care in children with heart disease. Springer-Verlag 1983;4(4):259–263. doi: 10.1007/BF02278871

34. Tong EM, Kools S. Health care transitions for adolescents with congenital heart disease: patient and family perspectives. The Nursing clinics of North America United States; 2004;39(4):727–40. doi: 10.1016/j.cnur.2004.07.011

35. Staveski SL, Zhelva B, Paul R, Conway R, Carlson A, Soma G, Kools S, Franck LS. Pediatric cardiac surgery Parent Education Discharge Instruction (PEDI) program: a pilot study. World journal for pediatric & congenital heart surgery United States; 2015;6(1):18–25. doi: 10.1177/2150135114554659

36. Sobierajski F, Storey K, Bird M, Anthony S, Pol S, Pidborochynski T, Balmer-Minnes D, Tharani AR, Power A, Khoury M, Cunningham C, Jeewa A, Conway J. Use of Photovoice to Explore Pediatric Patients with Hypertrophic Cardiomyopathy and their Parents’ Perceptions of a Heart-Healthy Lifestyle. American Heart Association Inc 2022;11(7). doi: 10.1161/JAHA.121.023572

37. Schumacher KR, Lee JM, Pasquali SK. Social media in paediatric heart disease: Professional use and opportunities to improve cardiac care. Cambridge University Press 2015;25(8):1584–1589. doi: 10.1017/S1047951115002292

38. Schmitt KRL, Sievers LK, Hütter A, Abdul-Khaliq H, Poryo M, Berger F, Bauer UMM, Helm PC, Pfitzer C. New Insights into the Education of Children with Congenital Heart Disease with and without Trisomy 21. Multidisciplinary Digital Publishing Institute (MDPI) 2023;59(1). doi: 10.3390/medicina59112001

39. Sanyahumbi A, Chiromo P, Chiume M. Education: The prevention of acute rheumatic fever and rheumatic heart disease in Malawi. Malawi Medical Journal 2019;31(3):223–224. doi: 10.4314/mmj.v31i3.9

40. Saidi A, Reiss J, Breitinger P, Black E, Paolillo J, Collins S. Web-based learning: Is it an effective method for educating pediatric residents about transition to adult subspecialty congenital heart disease care? [’Nova Biomedical Books’, ’US’] Hauppauge, NY, US; 2012;693–704. Available from: http://ovidsp.ovid.com/ovidweb.cgi?T=JS&PAGE=reference&D=psyc11&NEWS=N&AN=2013-16673-053

41. Saef J, SandeepTecson KM al R. Contributors to disease-specific health knowledge in adults with congenital heart disease: A correlational study. CONGENITAL HEART DISEASE 2018 Dec;13(6):967–977. doi: 10.1111/chd.12668

42. Sacks LD, Axelrod DM. Virtual reality in pediatric cardiology: Hype or hope for the future? Lippincott Williams and Wilkins 2020;35(1):37–41. doi: 10.1097/HCO.0000000000000694

43. Riley JP, Habibi H, Banya W, Gatzoulis MA, Lau-Walker M, Cowie MR. Education and support needs of the older adult with congenital heart disease. Journal of advanced nursing England; 2012;68(5):1050–60. doi: 10.1111/j.1365-2648.2011.05809.x

44. Ricci P, KonstantinosBouchard M and. Transition to adult care of young people with congenital heart disease: impact of a service on knowledge and self-care skills and correlates of a successful transition. EUROPEAN HEART JOURNAL-QUALITY OF CARE AND CLINICAL OUTCOMES 2023 Jun 21;9(4):351–357. doi: 10.1093/ehjqcco/qcad014

45. Penny, Daniel J. Speaking to children and their families about congenital heart disease: Ushering in a new era of healthcare literacy. Congenital heart disease United States; 2017;12(3):241. doi: 10.1111/chd.12474

46. Olderog-Hermiston E, Nowak A, Kanellis M, Olderog-Hermiston E J, Nowak A J, Kanellis M J. Practices and attitudes concerning oral health in pediatric cardiology clinics to prevent infective endocarditis. American Journal of Cardiology Philadelphia, Pennsylvania; 1998 Jun 15;81(1):1500–1502. doi: 10.1016/s0002-9149(98)00207-0

47. Nematollahi M, Bagherian B, Sharifi Z, Keshavarz F, Mehdipour‐Rabori R. Self‐care status in children with congenital heart disease: A mixed‐method study. Journal of Child & Adolescent Psychiatric Nursing 2020 May;33(2):77–84. doi: 10.1111/jcap.12265

48. Moons PdV, EBudts WdG, SElen J and. What do adult patients with congenital heart disease know about their disease, treatment, and prevention of complications? A call for structured patient education. HEART 2001 Jul;86(1):74–80. doi: 10.1136/heart.86.1.74

49. Mannarino CN, Michelson K, Jackson L, Paquette E, McBride ME. Post-operative discharge education for parent caregivers of children with congenital heart disease: A needs assessment. Cambridge University Press 2020;30(1):1788–1796. doi: 10.1017/S1047951120002759

50. Majnemer A, Mazer B, Lecker E, Carter AL, Limperopoulos C, Shevell M, Rohlicek C, Rosenblatt B, Tchervenkov C. Patterns of use of educational and rehabilitation services at school age for children with congenitally malformed hearts. Cardiol Young 2008;18(3):288–296. doi: 10.1017/S1047951108002114

51. Majnemer A, Dahan-Oliel N, Rohlicek C, Hatzigeorgiou S, Mazer B, Maltais DB, Schmitz N. Educational and rehabilitation service utilization in adolescents born preterm or with a congenital heart defect and at high risk for disability. Blackwell Publishing Ltd 2017;59(1):1056–1062. doi: 10.1111/dmcn.13520

52. Mackie AS, Islam S, Magill-Evans J, Rankin KN, Robert C, Schuh M, Nicholas D, Vonder Muhll I, McCrindle BW, Yasui Y, Rempel GR. Healthcare transition for youth with heart disease: a clinical trial. Heart (British Cardiac Society) England; 2014;100(1):1113–8. doi: 10.1136/heartjnl-2014-305748

53. Mackie AS, Rempel GR, Kovacs AH, Kaufman M, Rankin KN, Jelen A, Yaskina M, Sananes R, Oechslin E, Dragieva D, Mustafa S, Williams E, Schuh M, Manlhiot C, Anthony SJ, Magill-Evans J, Nicholas D, McCrindle BW. Transition Intervention for Adolescents With Congenital Heart Disease. Elsevier USA 2018;71(1):1768–1777. doi: 10.1016/j.jacc.2018.02.043

54. Lok SW, Menahem S. Children’s and adolescents’ understanding of their small ventricular septal defects. Pediatrics international : official journal of the Japan Pediatric Society Australia; 2012;54(6):824–8. doi: 10.1111/j.1442-200X.2012.03736.x

55. Lin Z-W, Liu J-F, Xie W-P, Zhang Q-L, Cao H, Chen Q. Performance of remote health education via WeChat to improve the pre-operative nutritional status of infants with non-restrictive ventricular septal defects: A prospective randomised controlled study. Journal of paediatrics and child health Australia; 2021;57(1):1666–1671. doi: 10.1111/jpc.15590

56. Lesch W, Specht K, Lux A, Frey M, Utens E, Bauer U. Disease-specific knowledge and information preferences of young patients with congenital heart disease. Cardiology in the young England; 2014;24(2):321–30. doi: 10.1017/S1047951113000413

57. Lee S, Lee J, Choi JY. The effect of a resilience improvement program for adolescents with complex congenital heart disease. SAGE Publications Inc 2017;16(4):290–298. doi: 10.1177/1474515116659836

58. Lapão LV, Correia A. Improving access to pediatric cardiology in Cape Verde via a collaborative international telemedicine service. IOS Press 2015;209(J):51–57. doi: 10.3233/978-1-61499-505-0-51

59. Lane B, Hanke SP, Giambra B, Madsen NL, Staveski SL. Development of a clinician-parent home care education intervention. Cambridge University Press 2019;29(1):1230–1235. doi: 10.1017/S1047951119001318

60. Kieu V, Sumski C, Cohen S, Reinhardt E, Axelrod DM, Handler SS. The Use of Virtual Reality Learning on Transition Education in Adolescents with Congenital Heart Disease. Springer 2023;44(8):1856–1860. doi: 10.1007/s00246-023-03292-w

61. Kassa A-M, Engvall G, Engstrand Lilja H. Young children with severe congenital malformations (VACTERL) expressed mixed feelings about their condition and worries about needles and anaesthesia. Acta paediatrica (Oslo, Norway : 1992) Norway; 2017;106(1):1694–1701. doi: 10.1111/apa.13973

62. Jackson JL, Tierney K, Daniels CJ, Vannatta K. Disease knowledge, perceived risk, and health behavior engagement among adolescents and adults with congenital heart disease. Heart & lung : the journal of critical care United States; 2015;44(1):39–44. doi: 10.1016/j.hrtlng.2014.08.009

63. Huang H-R, Chen C-W, Chen C-M, Yang H-L, Su W-J, Wang J-K, Tsai P-K. A positive perspective of knowledge, attitude, and practices for health-promoting behaviors of adolescents with congenital heart disease. SAGE Publications Inc 2018;17(3):217–225. doi: 10.1177/1474515117728609

64. Hill GD, Bingler M, McCoy AB, Oster ME, Uzark K, Bates KE. Improved National Outcomes Achieved in a Cardiac Learning Health Collaborative Based on Early Performance Level. The Journal of pediatrics United States; 2020;222:186–192. doi: 10.1016/j.jpeds.2020.03.014

65. Helm PC, Kempert S, Korten M-A, Lesch W, Specht K, Bauer UMM. Congenital heart disease patients’ and parents’ perception of disease-specific knowledge: Health and impairments in everyday life. Congenital heart disease United States; 2018;13(3):377–383. doi: 10.1111/chd.12581

66. Gordon BM, Lam TS, Bahjri K, Hashmi A, Kuhn MA. Utility of preprocedure checklists in the congenital cardiac catheterization laboratory. Congenital heart disease United States; 2014;9(2):131–7. doi: 10.1111/chd.12107

67. Gallagher A, Dagenais L, Doussau A, Décarie J-C, Materassi M, Gagnon K, Prud’homme J, Vobecky S, Poirier N, Carmant L. Significant motor improvement in an infant with congenital heart disease and a rolandic stroke: The impact of early intervention. Taylor and Francis Ltd 2017;20(3):165–168. doi: 10.3109/17518423.2015.1132280

68. Flocco SF, Dellafiore F, Caruso R, Giamberti A, Micheletti A, Negura DG, Piazza L, Carminati M, Chessa M. Improving health perception through a transition care model for adolescents with congenital heart disease. Lippincott Williams and Wilkins 2019;20(4):253–260. doi: 10.2459/JCM.0000000000000770

69. Feng R, Zhai B, Wang P, Song R. A comparative study of family centered nursing mode and routine clinical nursing mode on postoperative nursing of children with congenital heart disease. Pakistan Medical Association 2020;70(1):16–23. Available from: https://www.scopus.com/inward/record.uri?eid=2-s2.0-85096081652&partnerID=40&md5=715deb590b458fa03fb3eca880ad4119

70. de Hosson M, De Groote K, Wynendaele H, Mosquera LM, Goossens E, De Backer J. Preferences for disease-related information and transitional skills among adolescents with congenital heart disease in the early transitional stage. Springer Science and Business Media Deutschland GmbH 2023;182(9):3917–3927. doi: 10.1007/s00431-023-05020-1

71. Daily J, FitzGerald M, Downing K, King E, del Rey JG, Ittenbach R, Marino B. Important knowledge for parents of children with heart disease: parent, nurse, and physician views. Cardiology in the young England; 2016;26(1):61–9. doi: 10.1017/S1047951114002625

72. Chen S-S, Hou-TsanPai T-W. Intelligent Medical Interactive Educational System for Cardiovascular Disease. 2022;101. doi: 10.1007/978-3-031-08530-7_9

73. Charles S, Mackie AS, Rogers LG, McCrindle BW, Kovacs AH, Yaskina M, Williams E, Dragieva D, Mustafa S, Schuh M, Anthony SJ, Rempel GR. A Typology of Transition Readiness for Adolescents with Congenital Heart Disease in Preparation for Transfer from Pediatric to Adult Care. Journal of Pediatric Nursing Philadelphia, Pennsylvania; 2021 Sep;60:267–274. doi: 10.1016/j.pedn.2021.07.016

74. Calle ACF, Maria CandidaManso P. Going home after a child’s cardiac surgery: education for safe care. REVISTA BRASILEIRA DE ENFERMAGEM 2021;74(4). doi: 10.1590/0034-7167-2020-1163

75. Bellander T, Karlsson A-M. Patient participation and learning in medical consultations about congenital heart defects. Public Library of Science 2019;14(7). doi: 10.1371/journal.pone.0220136

76. Ahn J-A, Lee S, Choi JY. Comparison of coping strategy and disease knowledge in dyads of parents and their adolescent with congenital heart disease. Lippincott Williams and Wilkins 2014;29(6):508–516. doi: 10.1097/JCN.0000000000000090

77. Agarwal HS, Wolfram KB, Slayton JM, Saville BR, Cutrer WB, Bichell DP, Harris ZL, Barr FE, Deshpande JK. Template of patient-specific summaries facilitates education and outcomes in paediatric cardiac surgery units. Interact Cardiovasc Thorac Surg 2013;17(4):704–709. doi: 10.1093/icvts/ivt293

78. Woodward, Cathy S. Keeping children with congenital heart disease healthy. Journal of pediatric health care : official publication of National Association of Pediatric Nurse Associates & Practitioners United States; 2011;25(6):373–8. doi: 10.1016/j.pedhc.2011.03.007

79. Werner O, HamoudaLavastre K and. Factors influencing the participation of adolescents and young adults with a congenital heart disease in a transition education program: A prospective multicentre controlled study. PATIENT EDUCATION AND COUNSELING 2019 Dec;102(1):2223–2230. doi: 10.1016/j.pec.2019.06.023

80. Wang Q, Hay M, Clarke D, Menahem S. Associations between knowledge of disease, depression and anxiety, social support, sense of coherence and optimism with health-related quality of life in an ambulatory sample of adolescents with heart disease. Cambridge University Press 2014;24(1):126–133. doi: 10.1017/S1047951113000012

81. Veldtman GR, Matley SL, Kendall L, Quirk J, Gibbs JL, Parsons JM, Hewison J. Illness understanding in children and adolescents with heart disease. BMJ Publishing Group 2000;84(4):395–397. doi: 10.1136/heart.84.4.395

82. Uzark K, Messiter E, Rosenthal A. Promoting dental health care in children with congenital heart disease. Pediatr Nurs 1986;12(2):97–99. Available from: https://www.scopus.com/inward/record.uri?eid=2-s2.0-0022690098&partnerID=40&md5=d6a51c86a057248836635faccc29da44

83. Uzark, , K. Counseling adolescents with congenital heart disease. J Cardiovasc Nurs 1992;6(3):65–73. doi: 10.1097/00005082-199204000-00007

84. Suvarna RM, Rai K, Hegde AM. Oral health of children with congenital heart disease following preventive treatment. The Journal of clinical pediatric dentistry Singapore; 2011;36(1):93–8. doi: 10.17796/jcpd.36.1.h337135318140078

85. Newall F, Johnston L, Monagle P. Optimising anticoagulant education in the paediatric setting using a validated model of education. Patient Educ Couns 2008;73(2):384–388. doi: 10.1016/j.pec.2008.07.027

86. McPheeters, , Melissa. A single nurse-led educational session may facilitate transition from paediatric to adult healthcare for adolescents with heart disease. Evidence-based nursing England; 2015;18(3):83. doi: 10.1136/eb-2014-101957

87. Ladouceur M, Calderon J, Traore M, Cheurfi R, Pagnon C, Khraiche D, Bajolle F, Bonnet D. Educational needs of adolescents with congenital heart disease: Impact of a transition intervention programme. Archives of cardiovascular diseases Netherlands; 2017;110(5):317–324. doi: 10.1016/j.acvd.2017.02.001

88. Koerdt S, Hartz J, Hollatz S, Frohwitter G, Kesting MR, Ewert P, Oberhoffer R, Deppe H. Dental prevention and disease awareness in children with congenital heart disease. Springer Verlag 2018;22(3):1487–1493. doi: 10.1007/s00784-017-2256-2

89. Kluger N, Charlier P, Perciaccante A. Jan misugi in captain tsubasa as an educational example for children with congenital heart disease. Tech Science Press 2020;15(3):163–165. doi: 10.32604/CHD.2020.012394

90. Gaskin K, Kennedy F. Care of infants, children and adults with congenital heart disease. NLM (Medline) 2019;34(8):37–42. doi: 10.7748/ns.2019.e11405

91. Campos EFL, Perin L, Assmann M, Lucchese F, Pellanda LC. Knowledge about the disease and the practice of physical activity in children and adolescents with congenital heart disease. Arquivos Brasileiros de Cardiologia 2020;114(5):786–792. doi: 10.36660/abc.20180417

92. Alderman, , L.M. Congenital heart disease. More than child’s play. Nursing 2000;30(5):41–47. doi: 10.1097/00152193-200030050-00028

93. Acuna Mora M, Sparud-Lundin C, Bratt E-L, Moons P. Person-centred transition programme to empower adolescents with congenital heart disease in the transition to adulthood: a study protocol for a hybrid randomised controlled trial (STEPSTONES project). BMJ open England; 2017;7(4). doi: 10.1136/bmjopen-2016-014593
